# Linking morphological and molecular taxonomy for the identification of poultry house, soil, and nest dwelling mites in the Western Palearctic

**DOI:** 10.1038/s41598-019-41958-9

**Published:** 2019-04-08

**Authors:** Monica R. Young, María L. Moraza, Eddie Ueckermann, Dieter Heylen, Lisa F. Baardsen, Jose Francisco Lima-Barbero, Shira Gal, Efrat Gavish-Regev, Yuval Gottlieb, Lise Roy, Eitan Recht, Marine El Adouzi, Eric Palevsky

**Affiliations:** 1 Centre for Biodiversity Genomics, University of Guelph, Guelph, Canada; 2 Departamento de Biología Ambiental, Facultad de Ciencias, Universidad de Navarra, Pamplona, Spain; 3 Unit for Environmental Sciences and Management, Potchefstroom Campus, North-West University, Private Bag X6001, Potchefstroom, 2520 South Africa; 4 Interuniversity Institute for Biostatistics and Statistical Bioinformatics, Hasselt University, Diepenbeek, Belgium; 5 Department of Ecology and Evolutionary Biology, Princeton University, Princeton, NJ United States of America; 6 Evolutionary Ecology Group, University of Antwerp, Wilrijk, Belgium; 7 Health and Biotecnology Group (SABIO), Institute for Game and Wildlife Research (CSIC-UCLM), Ciudad Real, Spain; 8 Sabiotec, Ciudad Real, Spain; 9 Newe-Ya’ar Research Center, Agricultural Research Organization, Ramat Yishay, Israel; 10 The National Natural History Collections, The Hebrew University of Jerusalem, Jerusalem, Israel; 11 Koret School of Veterinary Medicine, The Hebrew University of Jerusalem, Rehovot, Israel; 12 UMR 5175 CEFE, CNRS – Université de Montpellier – Université Paul-Valéry Montpellier – EPHE, Route de Mende, 34199 Montpellier, Cedex 5 France; 13 Plant Protection and Inspection Services, Bet Dagan, Israel

## Abstract

Because of its ability to expedite specimen identification and species delineation, the barcode index number (BIN) system presents a powerful tool to characterize hyperdiverse invertebrate groups such as the Acari (mites). However, the congruence between BINs and morphologically recognized species has seen limited testing in this taxon. We therefore apply this method towards the development of a barcode reference library for soil, poultry litter, and nest dwelling mites in the Western Palearctic. Through analysis of over 600 specimens, we provide DNA barcode coverage for 35 described species and 70 molecular taxonomic units (BINs). Nearly 80% of the species were accurately identified through this method, but just 60% perfectly matched (1:1) with BINs. High intraspecific divergences were found in 34% of the species examined and likely reflect cryptic diversity, highlighting the need for revision in these taxa. These findings provide a valuable resource for integrative pest management, but also highlight the importance of integrating morphological and molecular methods for fine-scale taxonomic resolution in poorly-known invertebrate lineages.

## Introduction

DNA barcoding^1^ alleviates many of the challenges associated with morphological specimen identification by comparing short, standardized fragments of DNA – typically 648 bp of the cytochrome *c* oxidase I (COI) gene for animals – to a well-curated reference library. The success of this method relies on the presence of a clearly defined ‘barcode gap’, where intraspecific divergences are much more constrained than interspecific divergences. Its presence not only enables rapid specimen identification, but also facilitates species delineation through molecularly defined taxonomic units, a process automated through the barcode index number (BIN) system^2^. BINs correspond well with morphologically recognized species in lineages with well-curated taxonomy^2–4^ and can improve taxonomic resolution by elucidating hidden diversity^5,6^. Consequently, BINs are a powerful tool for characterizing diversity in poorly-known, hyperdiverse, invertebrates^7–9^, but have seen limited validation in these taxa.

The mites (Acari) may exceed one million species, but remain poorly known because of their small size and cryptic morphology^10^. While BIN–based surveys have expedited surveys of this hyperdiverse group^7,11,12^, the rapidly growing collection of mite barcodes generally lack lower-level taxonomy. For example, just 18% of the >12,400 mite BINs (from nearly 120,000 DNA barcode sequences) on the Barcode of Life Data System (BOLD, v4.boldsystems.org) are linked with a species name (accessed August 2018). Nonetheless, successful species delineation through DNA barcodes has been documented in several mite lineages, including the Ixodida^13^, Mesostigmata^14^, Sarcoptiformes^15^, and Trombidiformes^16^. DNA barcodes have also helped resolve issues like lumping due to cryptic morphology^17^, and splitting due to heteromorphy^18^. However, concordance between species and BINs has only been tested in a single mite lineage: medically important ticks from Canada^19^.

While many species of mites have detrimentally impacted human health and agriculture^20,21^, others are recognized for their benefits as biological control agents^22^. The poultry red mite (PRM; *Dermanyssus gallinae* (De Geer, 1778), for example, is a widespread pest with significant economic costs^23^. Since the PRM is now resistant to most acaricides, the need for novel biocontrol methods is greater than ever^24,25^. From this perspective, natural mite communities in soil and bird nests may provide novel predators for conservation biological control of the PRM, but have seen limited investigation^26–28^. In the present study we begin the development of a DNA barcode reference library for the identification of poultry litter, soil, and nest dwelling mites in the Western Palearctic. Specifically, we test the correspondence between BINs and traditionally recognized species, and analyze intraspecific divergences at COI to identify potentially cryptic taxa.

## Methods

### Specimen Collection and Preparation

**Figure 1:**
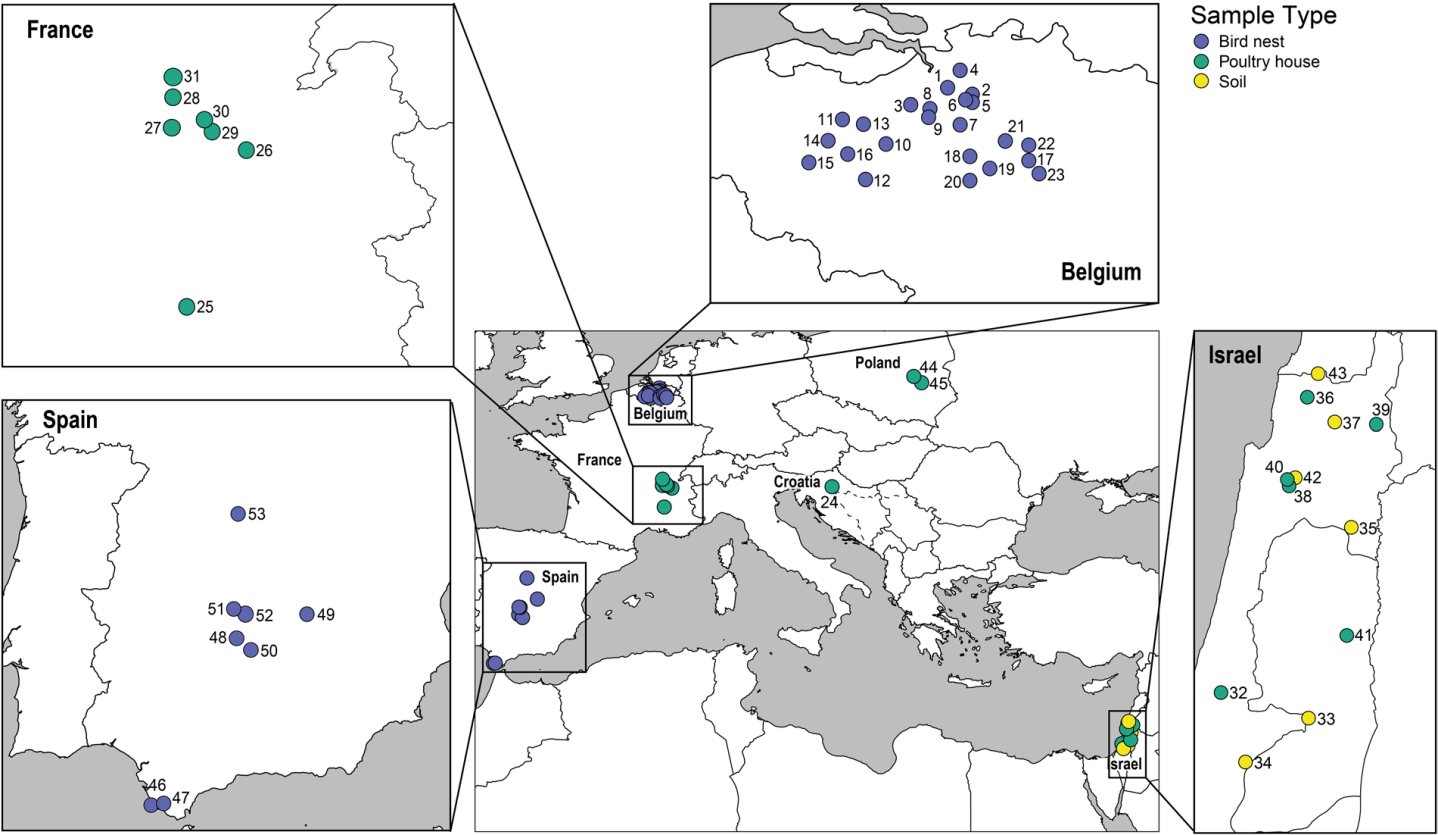
Map of the 53 sampling sites in seven countries across the Western Palearctic. The location markers correspond with site numbers specified in Table 1; sample type (bird nest, poultry house, soil) is indicated by the colour of the marker.

Samples of poultry litter and soil from the vicinity of poultry houses, as well as wild bird nests, were collected between 2015 and 2016 from 53 locations in Croatia, Belgium, France, Israel, Poland, and Spain (Fig. 1, Table 1). Mites were extracted from approximately 0.5 kg of substrate into 99% ethanol (EtOH) using modified Berlese-Tullgren funnels for five days. From each unique collection event (denoted by exact site and collection date), all mites, regardless of life stage or sex, were sorted to morphotype and identified to order using a standard stereomicroscope setup and keys in Krantz and Walter^29^. Up to five specimens per morphotype were selected for molecular analysis. Each specimen was imaged using a Leica DVM6 microscope and arrayed into a 96-well microplate (Eppendorf) containing 30 µL of 99% EtOH, with one blank well serving as a negative control. The museum identification code (Sample ID), collection details, order level taxonomy, and specimen images were uploaded to BOLD, available in the dataset DS-SMRPM through at 10.5883/DS-SMRPM.

**Table 1:** Summary of the 53 collection locations including the type of sample collected at each locality.

Site No.	Country	State/Province	Exact Site	Lat	Lon	Sample Type
1	Belgium	Antwerp	Antwerpen	51.1911	4.4267	Bird nest
2	Belgium	Antwerp	Boechout (Boshoek)	51.1241	4.5228	Bird nest
3	Belgium	Antwerp	Bornem	51.1106	4.2284	Bird nest
4	Belgium	Antwerp	Brasschaat	51.2718	4.4852	Bird nest
5	Belgium	Antwerp	Hove (Boshoek)	51.1367	4.5096	Bird nest
6	Belgium	Antwerp	Lint	51.1266	4.4933	Bird nest
7	Belgium	Antwerp	Mechelen	51.0229	4.4848	Bird nest
8	Belgium	Antwerp	Niel	51.1010	4.3409	Bird nest
9	Belgium	Antwerp	Puurs	51.0847	4.3244	Bird nest
10	Belgium	East Flanders	Aalst	50.9397	4.0578	Bird nest
11	Belgium	East Flanders	Destelbergen	51.0539	3.8203	Bird nest
12	Belgium	East Flanders	Gerardsbergen	50.7741	3.9422	Bird nest
13	Belgium	East Flanders	Kalken	51.0358	3.9221	Bird nest
14	Belgium	East Flanders	Merelbeke	50.9446	3.7177	Bird nest
15	Belgium	East Flanders	Oudenaarde	50.8456	3.6093	Bird nest
16	Belgium	East Flanders	Zottegem	50.8951	3.8262	Bird nest
17	Belgium	Flemish Brabant	Boutersem	50.8561	4.8739	Bird nest
18	Belgium	Flemish Brabant	Kortenberg	50.8739	4.5413	Bird nest
19	Belgium	Flemish Brabant	Oud-Heverlee	50.8198	4.6675	Bird nest
20	Belgium	Flemish Brabant	Overijse	50.7701	4.5389	Bird nest
21	Belgium	Flemish Brabant	Rotselaar	50.9451	4.7487	Bird nest
22	Belgium	Flemish Brabant	Tielt-Winge	50.9243	4.8641	Bird nest
23	Belgium	Flemish Brabant	Tienen	50.8045	4.9321	Bird nest
24	Croatia		Zagreb	45.8248	15.969	Poultry litter
25	France	Auvergne Rhones Alpes	Issirac	44.7233	5.0411	Poultry litter
26	France	Auvergne Rhones Alpes	Lhuis	45.7482	5.5416	Poultry litter
27	France	Auvergne Rhones Alpes	Mionnay	45.8948	4.9199	Poultry litter
28	France	Auvergne Rhones Alpes	Relevant	46.0897	4.9450	Poultry litter
29	France	Auvergne Rhones Alpes	Relevant	45.8791	5.2552	Poultry litter
30	France	Auvergne Rhones Alpes	Rignieux	45.9491	5.1788	Poultry litter
31	France	Auvergne Rhones Alpes	Saint Etienne du Bois	46.2330	4.9319	Poultry litter
32	Israel	Central Coastal Plain	Moshav Satria	31.8915	34.8403	Poultry litter
33	Israel	Jerusalem	Jerusalem	31.7947	35.2410	Soil
34	Israel	Jerusalem	Nehusha	31.6284	34.9523	Soil
35	Israel	Northern	Bét Alfa	32.5176	35.4364	Soil
36	Israel	Northern	'En Ya’aqov	33.0093	35.2352	Poultry litter
37	Israel	Northern	Kammon	32.9154	35.3608	Soil
38	Israel	Northern	Kefar Yehoshua'	32.6747	35.1519	Poultry litter
39	Israel	Northern	Korazim	32.9070	35.5506	Poultry litter
40	Israel	Northern	New é Ya’ar	32.7056	35.1801	Soil
41	Israel	Northern	Ramat Zevi	32.1079	35.4158	Poultry litter
42	Israel	Northern	Sede Ya’aqov	32.6989	35.1439	Poultry litter
43	Israel	Northern	Zar’it	33.0985	35.2847	Soil
44	Poland	Masovian	Deba	51.4387	22.1781	Poultry litter
45	Poland	Masovian	Zygmunty	51.7810	21.6713	Poultry litter
46	Spain	Andalusia	Castilnovo	36.2530	−6.0803	Bird nest
47	Spain	Andalusia	La Barca de Vejer	36.2605	−5.9613	Bird nest
48	Spain	Castilla-La Mancha	Abenojar	38.8958	−4.4366	Bird nest
49	Spain	Castilla-La Mancha	Alcazar de San Juan	39.3899	−3.2109	Bird nest
50	Spain	Castilla-La Mancha	Almodovar del Campo	38.7312	−4.1880	Bird nest
51	Spain	Castilla-La Mancha	Cabaneros National Park	39.2852	−4.3392	Bird nest
52	Spain	Castilla-La Mancha	El Rostro	39.2971	−4.4165	Bird nest
53	Spain	Comunidad de Madrid	Rascafria	40.8717	−3.8982	Bird nest

### Molecular Analysis

The specimens were sequenced for the barcode region of COI using standard invertebrate DNA extraction^30,31^, amplification^32^ and sequencing protocols^33^ at the Canadian Centre for DNA barcoding (CCDB; http://ccdb.ca/). However, DNA extraction was modified following Porco *et al*.^34^ to facilitate the recovery of voucher specimens. A cocktail (1:1 ratio) of LepF1/LepRI^1^ and LCO1490/HCO2198^35^ primers were chosen to amplify and sequence a 652 bp fragment of DNA from the barcode region of COI because of their prior success in a broad array of mite taxa^11^. The DNA extracts were archived in −80 °C freezers at the Centre for Biodiversity Genomics (CBG; biodiversitygenomics.net), and the specimen vouchers were stored in 95% EtOH and returned to the Newe-Ya’ar Research Center and the Centre d’Ecologic Functionnelle & Evolutine for morphological preparations.

The forward and reverse chromatograms were assembled into consensus sequences for each specimen and edited using CodonCode Aligner v. 4.2.7 and uploaded to BOLD. Each sequence meeting minimum quality criteria (≥500 base pairs, <1% ambiguous nucelotides, free of contamination and stop codons) was assigned a BIN by BOLD. The sequences were further validated by inspecting their placement in a Neighbor-Joining tree (K2P distance model, BOLD alignment) and corresponding specimen images using the ‘Taxon ID Tree’ function in BOLD (Supplementary Figs 1 and 2). Taxa with unexpected placement in the tree (i.e. conflicting identifications within a cluster, conspecifics forming outgroups, etc.) were blasted against all barcode records on BOLD using the ‘Identification Engine’ tool whereupon instances of contamination (i.e. bacteria, Insecta, etc.) were flagged and filtered from the reference library.

### Specimen Identification

Following BIN assignment, up to five vouchers per BIN were prepared for light microscopy by either mounting the specimens directly into Hoyer’s medium, or in the case of Oribatida, placing the specimen in lactic acid on a cavity slide. Since the specimens were sufficiently cleared during the tissue lysis stage of DNA extraction, the typical clearing procedures were not necessary. All remaining vouchers were prepared for SEM imaging on a Hitachi TM3000 TableTop Scanning Electron Microscope, with standard drying and coating procedures.

Each specimen was identified to the lowest possible level of taxonomy, and compared to identifications of other members of the same BIN. Some specimens were not slide mounted because of redundancy, or morphologically identified when precluded by their life stage, sex or voucher quality, and were thus assigned the lowest level of taxonomy in agreement with other members in the BIN. Specimens identified in this way were denoted by ‘BIN Taxonomy Match’ in the Identification Method field.

### Data Analysis

Sampling completeness was assessed by constructing a BIN accumulation curve and by estimating total BIN richness using the incidence coverage estimator (ICE) in EstimateS^36^. Maximum intraspecific and minimum interspecific p-distances were calculated for all morphologically identified specimens using the ‘Barcode Gap Analysis’ tool on BOLD. Species correspondence with BINs were characterized by one of four categories: matches (perfect correspondence between one species and one BIN), splits (one species is represented by more than one BIN), merges (two or more species are assigned to a single BIN), and mixtures (a combination of splits and merges) as described in Ratnasingham and Hebert^2^.

## Results

### Sequence Recovery

Barcode compliant sequences were recovered from 298 of the 652 specimens analysed, with an overall PCR success rate of 76.5% and sequencing success rate of 45.7%. Success varied greatly among the major lineages. PCR success, for example, ranged from a high of 85% in the Trombidiformes, to a low of 45% in the Astigmatina (Sarcoptiformes). Sequencing success, on the other hand, ranged from a high of 56% in the Mesostigmata to a low of 0% in the Astigmatina (Sarcoptiformes) and Opilioacarida (Table 2). Non-target amplification was detected in 28 sequences, including cross-mite contamination, insects, and occasionally bacteria. These sequences were flagged on BOLD, removed from the BOLD identification engine, and excluded from subsequent analyses.

**Table 2:** Summary of the number of specimens analysed, with the number of PCR products and barcode compliant sequences generated for each order.

Taxon	Specimens	PCR Products	Sequences
Mesostigmata	456	373 (81.8%)	254 (55.7%)
Opilioacarida	4	3 (75.0%)	0 (0.0%)
Sarcoptiformes: Astigmatina	106	48 (45.3%)	0 (0.0%)
Sarcoptiformes: Oribatida	10	10 (100%)	4 (4.0%)
Trombidiformes	76	65 (85.5%)	40 (52.6%)
Total	652	499 (76.5%)	298 (45.7%)

Success rates are provided in brackets.

### DNA Barcode Reference Library and Sample Completeness

**Figure 2:**
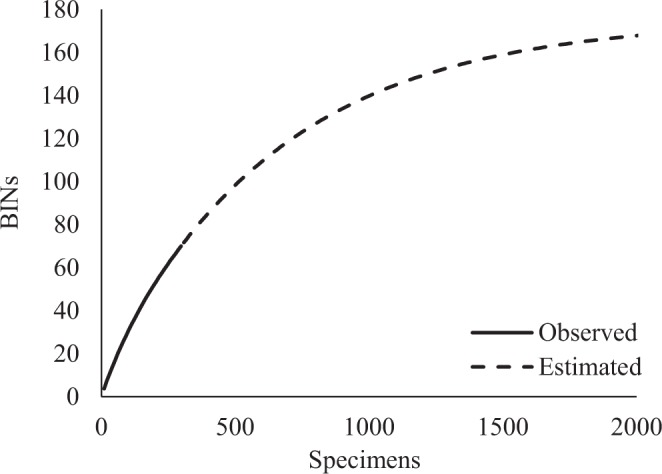
The observed (solid line) and estimated (dashed line) accumulation of BINs with increasing sample size for the 298 specimens with BIN assignments.

Minimum quality requirements for BIN assignment were met by 298 sequences representing 70 BINs in total ($\bar{x}\,=\,$4.2 specimens/BIN). Of these 70 BINs, 48 (68.6%) were morphologically identified to the species level, while genus was the lowest identification for six BINs (8.6%), family for 15 BINs (21.4%), and one BIN was identified only to the order level (1.4%). In total, 35 species, 27 genera, 24 families, and three orders were identified in our barcode reference library (Table 3). The slope of the BIN accumulation curve remains steep, indicating incomplete sampling of the fauna (Fig. 2), and the estimate of total BIN richness was more than double the current observations (ICE = 172 BINs).

**Table 3:** Breakdown of the 652 specimens analysed including the number of sequences with BIN assignments and summary of BINs for each taxon. Species are characterized into BIN categories with estimates of intra- and interspecific distances.

Order	Family	Genus/Species	Specimens	Specimens with BINs	BINs	BIN Category	Maximum Intraspecific P-distance (%)	Minimum Interspecific P-distance (%)
Mesostigmata		Mesostigmata spp.	167	2	1			
	Ameroseiidae	*Ameroseius eumorphus* Bregetova, 1077	5	5	1	Match	1.87	28.92
		*Ameroseius macrochelae* (Westerboer, 1963)	1	1	1	Singleton		
		*Ameroseius* sp.	2	2	1			
	Ascidae	Ascidae sp.	1	1	1			
	Blattisociidae	*Lasioseius floridensis* Berlese, 1916	9	8	2	Split	19.36	24.42
	Dermanyssidae	*Dermanyssus carpathicus* Zeman, 1979	5	5	1	Match	1.15	19.29
		*Dermanyssus gallinae* (De Geer, 1778)	7	7	1	Match	2.99	19.29
	Digamasellidae	Digamasellidae sp.	4	4	1			
		*Dendrolaelaps longisculus* (Leitner, 1949)	8	8	1	Mixture	0.19	0
		Dendrolaelaps presepum* (Berlese, 1918)	19	15	3	Mixture	24.70	0
	Laelapidae	Laelapidae spp.	3	1	1			
		Androlaelaps casalis* (Berlese, 1887)	19	12	2	Split	33.63	20.02
		*Androlaelaps* sp.	1	1	1			
		*Gaeolaelaps aculeifer* (Canestrini 1883)	5	5	1	Match	0	22.11
		*Stratiolaelaps scimitus* (Berlese, 1892)	4	4	1	Match	0	20.02
	Macrochelidae	Macrocheles matrius* (Hull, 1925)	5	5	1	Match	0.15	22.52
		Macrocheles merdarius* (Berlese, 1889)	16	15	2	Split	21.89	23.16
		Macrocheles muscaedomesticae* (Scopoli, 1772)	21	21	1	Match	0.46	22.52
		*Macrocheles penicilliger* (Berlese, 1904)	5	5	2	Split	32.21	25.42
		*Macrocheles scutatiformis* Petrova, 1967	1	1	1	Singleton		
	Macronyssidae	*Ornithonyssus sylviarum* (Canestrini & Fanzago, 1877)	5	5	1	Match	0	25.89
	Melicharidae	*Proctolaelaps* sp.	4	4	1			
		Proctolaelaps nr. parascolyti *Costa, 1963	7	7	4	Mixture	21.17	0
		*Proctolaelaps pygmaeus* (Müller, 1859)	5	6	2	Mixture	3.60	0
		*Proctolaelaps scolyti* Evans, 1958	8	8	2	Split	16.95	3.15
	Parasitidae	*Cologamasus* sp.	1	1	1			
		Gamasodes spiniger* (Oudemans, 1936)	13	13	1	Match	2.67	17.02
		Parasitus fimetorum* Hyatt, 1980	16	15	2	Split	17.40	17.32
		*Parasitus hyalinus* (Willmann, 1949)	12	12	1	Match	1.24	20.41
		*Poecilochirus carabi* G. Canestrini & R. Canestrini, 1882	7	7	1	Match	1.60	17.32
		*Vulgarogamasus burchanensis* (Oudemans, 1903)	13	13	1	Match	0.77	17.02
	Polyaspidae	*Uroseius* sp.	2	2	1			
	Rhodacaridae	*Protogamasellopsis corticalis* Evans &Purvis, 1987	11	11	3	Split	27.38	22.74
		*Rhodacarellus silesiacus* Willmann, 1935	3	3	2	Split	2.97	17.52
	Trematuridae	Trematuridae sp.	1	1	1			
		*Nenteria floralis* Karg, 1986	5	—	—			
		*Trichouropoda orbicularis* (C.L. Koch, 1839)	4	4	1	Match	0	10.03
		*Trichouropoda ovalis* (C.L. Koch, 1839)	4	4	1	Match	0.16	17.90
	Urodinychidae	Uroobovella fimicola* (Berlese, 1903)	8	6	1	Match	1.08	22.96
		Uroobovella marginata* (C. L. Koch, 1839)	13	1	1	Singleton		
	Uropodidae	*Uropoda orbicularis* (Müller, 1776)	4	4	1	Match	1.46	10.03
Opilioacarida		Opilioacarida sp.	4	—	—			
Sarcoptiformes		Astigmatina spp.	65	—	—			
		Oribatida spp.	47	—	—			
	Oppiidae	Oppiidae sp.	4	4	1			
Trombidiformes		Trombidiformes spp.	35	—	—			
	Anystidae	Anystidae sp.	1	1	1			
	Bdellidae	Bdellidae spp.	8	8	4			
	Cheyletidae	*Cheletomorpha lepidopterorum* (Shaw, 1794)	2	2	1	Match	0	28.92
		*Cheyletus bidentatus* Fain and Nadchatram, 1980	14	14	1	Match	0.54	3.47
		Cheyletus malaccensis* (Oudemans, 1903)	5	5	1	Match	0.77	3.47
	Cunaxidae	Cunaxidae spp.	4	4	3			
	Erythracaridae	Erythracaridae sp.	1	1	1			
	Eupodidae	Eupodidae sp.	1	—	—			
	Scutacaridae	Scutacaridae sp.	1	1	1			
	Tetranychidae	*Tetranychus urticae* (C.L. Koch, 1833)	1	1	1	Singleton		
	Tydeidae	Tydeidae sp.	3	3	1			

The species previously associated with the poultry red mite are denoted by asterisks (*).

### Barcode Gap and BIN Analysis

**Figure 3:**
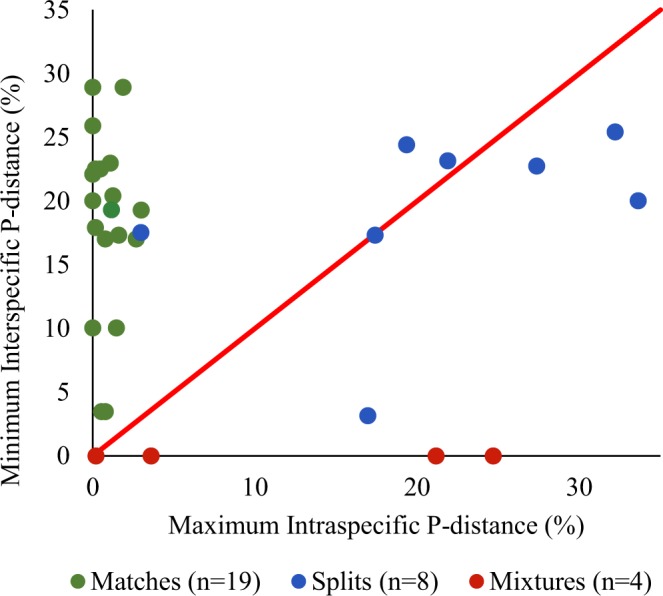
Comparison of maximum intraspecific and minim interspecific divergences (p-distances) of the 35 morphologically identified species. Data points are colourized based on species correspondence with BINs, and the diagonal red line indicates the 1:1 ratio of divergences. The barcode gap is present in species that fall above the line, and absent in those below.

Of the 35 morphologically identified species with BINs, 19 (61%) perfectly corresponded with BIN assignments, while eight (26%) resulted in BIN splits, and two cases of BIN mixtures affecting four species (13%) were detected (Fig. 3, Table 3). The barcode gap analysis revealed nine species in which maximum interspecific p-distance exceeded minimum intraspecific p-distance (Fig. 3), all of which were involved in BIN splits or mixtures. Maximum intraspecific p-distances averaged 7.7%, and dropped to 0.9% when BIN splits and mixtures were excluded from analyses.

## Discussion

Through the integration of morphological and molecular taxonomic methods, we provide DNA barcode coverage for 35 described species and 70 mite BINs from soil, bird nest, and poultry house-associated assemblages in the Western Palearctic. The integrity of most vouchers was sufficiently maintained for morphological identification, and SEM imaging of diagnostic characters (see the following BIN page for example: BOLD:ADA3054). While only 13 of these species have been previously associated with the poultry red mite^27,37^, additional species are undoubtedly present in our dataset but remain undetected because of low sequencing success combined with several BINs lacking identifications. Our failure to generate any sequences for Astigmatina (Sarcoptiformes) may be explained by low primer affinity, considering amplification rates were also lowest in this group. Primer affinity, however, does not justify the low successes in other lineages with higher amplification rates. Comparable methods, for example, have yielded much higher successes (77%) among soil and leaf litter mites (including Astigmatina) from subarctic Canada^11^, demonstrating the broad applicability of these primers among a diverse array of taxa. Since 40% of the amplification products generated uninterpretable chromatograms, poor quality DNA template may be responsible for low sequencing successes among taxa.

The concordance between BINs and mite species was much lower than in some well-studied invertebrates (e.g. perfect concordance in 92% of beetles^4^ and ticks^19^). However, similar concordance levels have been reported for many taxa including geometrid moths^38^ (67%), true bugs^39^ (70%), and spiders^5^ (54%). Low concordance is mainly driven by species with large intraspecific divergences (>3% p-distance) resulting in the assignment of two or more BINs. While this does not preclude accurate barcode-based identification, it highlights potentially cryptic species because most BIN splits formed widely separated clades (e.g. >15% p-distance) lacking intermediate haplotypes. In fact, 16S and 18S rRNA gene topologies for *Androlaelaps casalis* (Berlese, 1887) and *Proctolaelaps scolyti* Evans, 1958 were congruent with BIN splits, further supporting our cryptic species hypothesis in these taxa^27^. *Rhodacarellus silesiacus* Willmann, 1936, on the other hand, also formed two distinct but narrowly separated clades (<3% divergence), with divergences similar to those in species with concordant BINs (e.g. *Dermanyssus gallinae* and *Gamasodes spiniger* (Oudemans, 1936)), such that additional sampling may reveal intermediate haplotypes causing the BINs to collapse into one^2^.

More problematic for the barcode based identification of mites are the two cases of shared barcodes confounded by BIN splits (BIN mixtures) affecting four species: *Dendrolaelaps longiusculus* (Leitner, 1949)/D*. presepum* (Berlese, 1918), and *Proctolaelaps parascolyti* Costa, 1963/*P. pygmaeus* (Müller, 1859). Since multiple species are assigned to the same BIN, mixtures impede accurate identifications, but may also represent taxonomic errors^2^. Misidentification is unlikely, since procedures were in place to evaluate and correct such errors. However, both cases of BIN mixtures involve closely allied congenerics which may be subjected to hybridization or incomplete lineage sorting^40^. Given the large intraspecific divergences observed, though, a more probable explanation is the presence of cryptic diversity compounded by inadequate species descriptions. Future work should scrutinize the morphology of genetic clusters from both mixtures and splits for more effective characters to discriminate these potentially cryptic species.

This study represents the first step towards development of a DNA barcode reference library for the identification of poultry litter, soil, and nest dwelling mites from the Western Palearctic, which may in turn reveal natural enemies key to the control of PRM. Although sequencing success rates should be improved, we demonstrate that nearly 80% of the species analysed can be accurately identified through DNA barcodes. Our BIN analysis, however, indicates a high proportion of cryptic diversity and some potential taxonomic confusion. This method consequently presents a powerful tool not only for the identification of unknown specimens, but as the foundation for integrative taxonomy and diversity estimation in hyperdiverse invertebrates such as mites.

## Supplementary information

Suuplementary Information

## Data Availability

All specimen and sequence data is available in the BOLD dataset DS-SMRPM through the following, 10.5883/DS-SMRPM. Valid sequences were also deposited in GenBank under the following accessions: MH983560-MH983861.
