# Supplementary material for: Linking morphological and molecular taxonomy for the identification of poultry house, soil, and nest dwelling mites in the Western Palearctic
Source: Sci Rep. 2019 Apr 8;9:5784. doi: 10.1038/s41598-019-41958-9 (PMC6453913; doi:10.1038/s41598-019-41958-9)
Supplement: Supplementary file 1 — Suuplementary Information [file 41598_2019_41958_MOESM1_ESM.pdf]

Linking morphological and molecular taxonomy for the identification of poultry house, soil, and nest dwelling mites in the Western Palearctic

Monica R. Young, María L. Moraza, Eddie Ueckermann, Dieter Heylen, Lisa F. Baardsen, Jose F. Lima-Barbero, Shira Gal, Efrat Gavish-Regev, Yuval Gottlieb, Lise Roy, Eitan Recht, Marine El Adouzi, and Eric Palevsky

Supplementary Information

Supplementary Figure 1: Neighbour-joining taxon ID tree for all barcode compliant mite sequences, with nodes colorized by family.

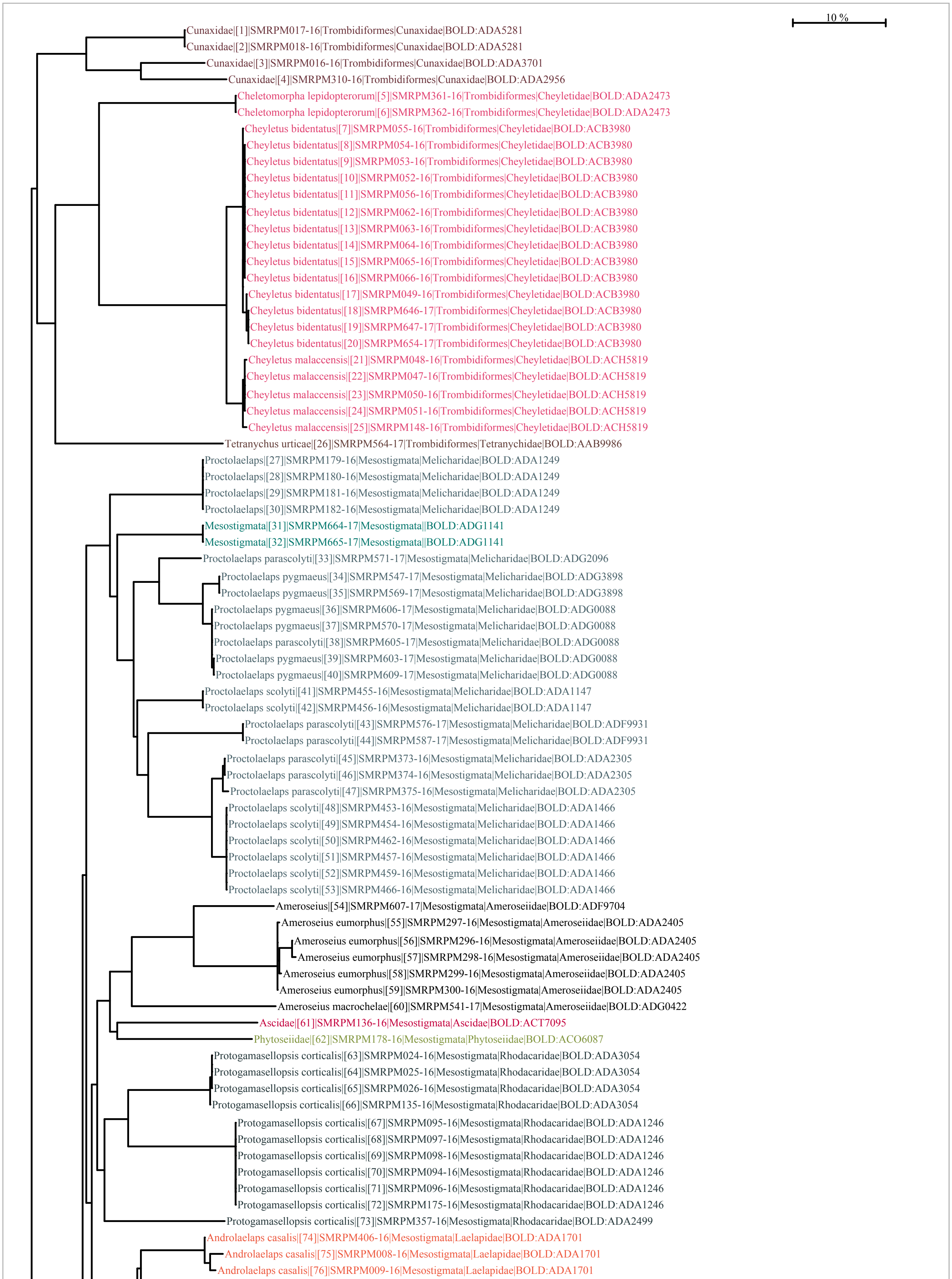

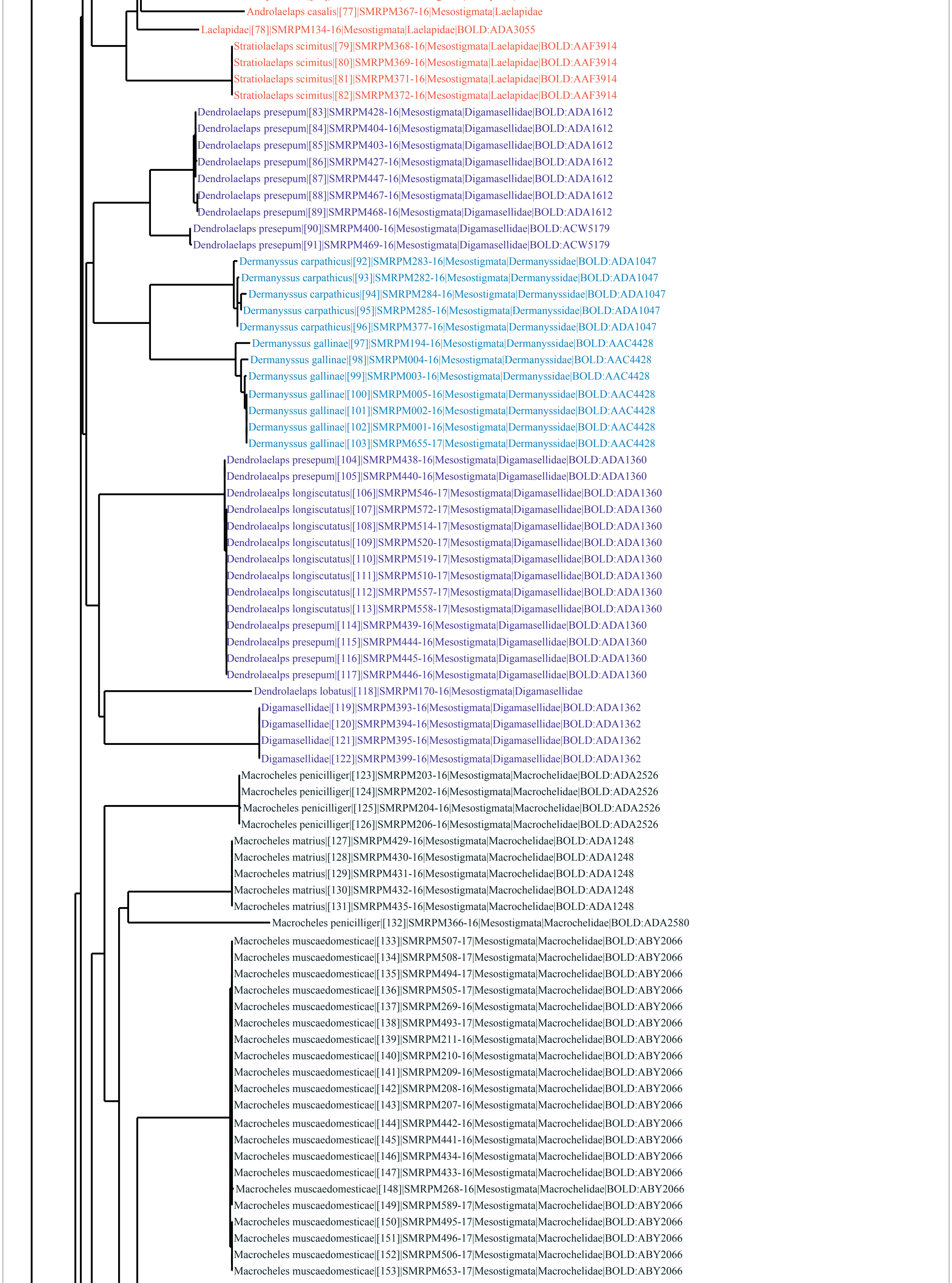
Androlaelaps casalis|[77]|SMRPM367-16|Mesostigmata|Laelapidae
Laelapidae|[78]|SMRPM134-16|Mesostigmata|Laelapidae|BOLD:ADA3055
Stratiolaelaps scimitus|[79]|SMRPM368-16|Mesostigmata|Laelapidae|BOLD:AAF3914
Stratiolaelaps scimitus|[80]|SMRPM369-16|Mesostigmata|Laelapidae|BOLD:AAF3914
Stratiolaelaps scimitus|[81]|SMRPM371-16|Mesostigmata|Laelapidae|BOLD:AAF3914
Stratiolaelaps scimitus|[82]|SMRPM372-16|Mesostigmata|Laelapidae|BOLD:AAF3914
Dendrolaelaps presepum|[83]|SMRPM428-16|Mesostigmata|Digamasellidae|BOLD:ADA1612
Dendrolaelaps presepum|[84]|SMRPM404-16|Mesostigmata|Digamasellidae|BOLD:ADA1612
Dendrolaelaps presepum|[85]|SMRPM403-16|Mesostigmata|Digamasellidae|BOLD:ADA1612
Dendrolaelaps presepum|[86]|SMRPM427-16|Mesostigmata|Digamasellidae|BOLD:ADA1612
Dendrolaelaps presepum|[87]|SMRPM447-16|Mesostigmata|Digamasellidae|BOLD:ADA1612
Dendrolaelaps presepum|[88]|SMRPM467-16|Mesostigmata|Digamasellidae|BOLD:ADA1612
Dendrolaelaps presepum|[89]|SMRPM468-16|Mesostigmata|Digamasellidae|BOLD:ADA1612
Dendrolaelaps presepum|[90]|SMRPM400-16|Mesostigmata|Digamasellidae|BOLD:ACW5179
Dendrolaelaps presepum|[91]|SMRPM469-16|Mesostigmata|Digamasellidae|BOLD:ACW5179
Dermanyssus carpathicus|[92]|SMRPM283-16|Mesostigmata|Dermanyssidae|BOLD:ADA1047
Dermanyssus carpathicus|[93]|SMRPM282-16|Mesostigmata|Dermanyssidae|BOLD:ADA1047
Dermanyssus carpathicus|[94]|SMRPM284-16|Mesostigmata|Dermanyssidae|BOLD:ADA1047
Dermanyssus carpathicus|[95]|SMRPM285-16|Mesostigmata|Dermanyssidae|BOLD:ADA1047
Dermanyssus carpathicus|[96]|SMRPM377-16|Mesostigmata|Dermanyssidae|BOLD:ADA1047
Dermanyssus gallinae|[97]|SMRPM194-16|Mesostigmata|Dermanyssidae|BOLD:AAC4428
Dermanyssus gallinae|[98]|SMRPM004-16|Mesostigmata|Dermanyssidae|BOLD:AAC4428
Dermanyssus gallinae|[99]|SMRPM003-16|Mesostigmata|Dermanyssidae|BOLD:AAC4428
Dermanyssus gallinae|[100]|SMRPM005-16|Mesostigmata|Dermanyssidae|BOLD:AAC4428
Dermanyssus gallinae|[101]|SMRPM002-16|Mesostigmata|Dermanyssidae|BOLD:AAC4428
Dermanyssus gallinae|[102]|SMRPM001-16|Mesostigmata|Dermanyssidae|BOLD:AAC4428
Dermanyssus gallinae|[103]|SMRPM655-17|Mesostigmata|Dermanyssidae|BOLD:AAC4428
Dendrolaelaps presepum|[104]|SMRPM438-16|Mesostigmata|Digamasellidae|BOLD:ADA1360
Dendrolaealps presepum|[105]|SMRPM440-16|Mesostigmata|Digamasellidae|BOLD:ADA1360
Dendrolaealps longiscutatus|[106]|SMRPM546-17|Mesostigmata|Digamasellidae|BOLD:ADA1360
Dendrolaealps longiscutatus|[107]|SMRPM572-17|Mesostigmata|Digamasellidae|BOLD:ADA1360
Dendrolaealps longiscutatus|[108]|SMRPM514-17|Mesostigmata|Digamasellidae|BOLD:ADA1360
Dendrolaealps longiscutatus|[109]|SMRPM520-17|Mesostigmata|Digamasellidae|BOLD:ADA1360
Dendrolaealps longiscutatus|[110]|SMRPM519-17|Mesostigmata|Digamasellidae|BOLD:ADA1360
Dendrolaealps longiscutatus|[111]|SMRPM510-17|Mesostigmata|Digamasellidae|BOLD:ADA1360
Dendrolaealps longiscutatus|[112]|SMRPM557-17|Mesostigmata|Digamasellidae|BOLD:ADA1360
Dendrolaealps longiscutatus|[113]|SMRPM558-17|Mesostigmata|Digamasellidae|BOLD:ADA1360
Dendrolaealps presepum|[114]|SMRPM439-16|Mesostigmata|Digamasellidae|BOLD:ADA1360
Dendrolaealps presepum|[115]|SMRPM444-16|Mesostigmata|Digamasellidae|BOLD:ADA1360
Dendrolaealps presepum|[116]|SMRPM445-16|Mesostigmata|Digamasellidae|BOLD:ADA1360
Dendrolaealps presepum|[117]|SMRPM446-16|Mesostigmata|Digamasellidae|BOLD:ADA1360
Dendrolaelaps lobatus|[118]|SMRPM170-16|Mesostigmata|Digamasellidae
Digamasellidae|[119]|SMRPM393-16|Mesostigmata|Digamasellidae|BOLD:ADA1362
Digamasellidae|[120]|SMRPM394-16|Mesostigmata|Digamasellidae|BOLD:ADA1362
Digamasellidae|[121]|SMRPM395-16|Mesostigmata|Digamasellidae|BOLD:ADA1362
Digamasellidae|[122]|SMRPM399-16|Mesostigmata|Digamasellidae|BOLD:ADA1362
Macrocheles penicilliger|[123]|SMRPM203-16|Mesostigmata|Macrochelidae|BOLD:ADA2526
Macrocheles penicilliger|[124]|SMRPM202-16|Mesostigmata|Macrochelidae|BOLD:ADA2526
Macrocheles penicilliger|[125]|SMRPM204-16|Mesostigmata|Macrochelidae|BOLD:ADA2526
Macrocheles penicilliger|[126]|SMRPM206-16|Mesostigmata|Macrochelidae|BOLD:ADA2526
Macrocheles matrius|[127]|SMRPM429-16|Mesostigmata|Macrochelidae|BOLD:ADA1248
Macrocheles matrius|[128]|SMRPM430-16|Mesostigmata|Macrochelidae|BOLD:ADA1248
Macrocheles matrius|[129]|SMRPM431-16|Mesostigmata|Macrochelidae|BOLD:ADA1248
Macrocheles matrius|[130]|SMRPM432-16|Mesostigmata|Macrochelidae|BOLD:ADA1248
Macrocheles matrius|[131]|SMRPM435-16|Mesostigmata|Macrochelidae|BOLD:ADA1248
Macrocheles penicilliger|[132]|SMRPM366-16|Mesostigmata|Macrochelidae|BOLD:ADA2580
Macrocheles muscaedomesticae|[133]|SMRPM507-17|Mesostigmata|Macrochelidae|BOLD:ABY2066
Macrocheles muscaedomesticae|[134]|SMRPM508-17|Mesostigmata|Macrochelidae|BOLD:ABY2066
Macrocheles muscaedomesticae|[135]|SMRPM494-17|Mesostigmata|Macrochelidae|BOLD:ABY2066
Macrocheles muscaedomesticae|[136]|SMRPM505-17|Mesostigmata|Macrochelidae|BOLD:ABY2066
Macrocheles muscaedomesticae|[137]|SMRPM269-16|Mesostigmata|Macrochelidae|BOLD:ABY2066
Macrocheles muscaedomesticae|[138]|SMRPM493-17|Mesostigmata|Macrochelidae|BOLD:ABY2066
Macrocheles muscaedomesticae|[139]|SMRPM211-16|Mesostigmata|Macrochelidae|BOLD:ABY2066
Macrocheles muscaedomesticae|[140]|SMRPM210-16|Mesostigmata|Macrochelidae|BOLD:ABY2066
Macrocheles muscaedomesticae|[141]|SMRPM209-16|Mesostigmata|Macrochelidae|BOLD:ABY2066
Macrocheles muscaedomesticae|[142]|SMRPM208-16|Mesostigmata|Macrochelidae|BOLD:ABY2066
Macrocheles muscaedomesticae|[143]|SMRPM207-16|Mesostigmata|Macrochelidae|BOLD:ABY2066
Macrocheles muscaedomesticae|[144]|SMRPM442-16|Mesostigmata|Macrochelidae|BOLD:ABY2066
Macrocheles muscaedomesticae|[145]|SMRPM441-16|Mesostigmata|Macrochelidae|BOLD:ABY2066
Macrocheles muscaedomesticae|[146]|SMRPM434-16|Mesostigmata|Macrochelidae|BOLD:ABY2066
Macrocheles muscaedomesticae|[147]|SMRPM433-16|Mesostigmata|Macrochelidae|BOLD:ABY2066
Macrocheles muscaedomesticae|[148]|SMRPM268-16|Mesostigmata|Macrochelidae|BOLD:ABY2066
Macrocheles muscaedomesticae|[149]|SMRPM589-17|Mesostigmata|Macrochelidae|BOLD:ABY2066
Macrocheles muscaedomesticae|[150]|SMRPM495-17|Mesostigmata|Macrochelidae|BOLD:ABY2066
Macrocheles muscaedomesticae|[151]|SMRPM496-17|Mesostigmata|Macrochelidae|BOLD:ABY2066
Macrocheles muscaedomesticae|[152]|SMRPM506-17|Mesostigmata|Macrochelidae|BOLD:ABY2066
Macrocheles muscaedomesticae|[153]|SMRPM653-17|Mesostigmata|Macrochelidae|BOLD:ABY2066

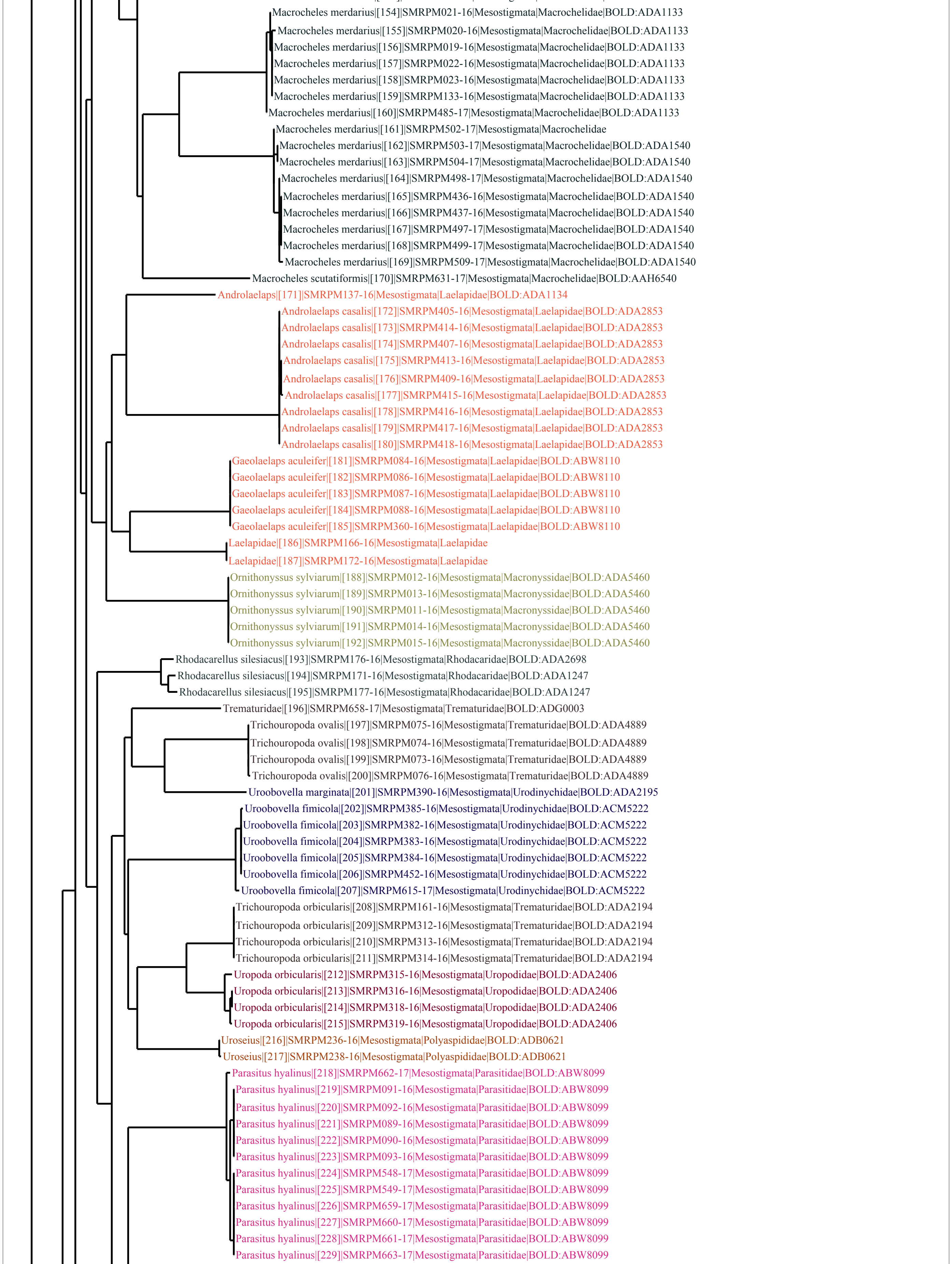
Macrocheles merdarius|[154]|SMRPM021-16|Mesostigmata|Macrochelidae|BOLD:ADA1133
Macrocheles merdarius|[155]|SMRPM020-16|Mesostigmata|Macrochelidae|BOLD:ADA1133
Macrocheles merdarius|[156]|SMRPM019-16|Mesostigmata|Macrochelidae|BOLD:ADA1133
Macrocheles merdarius|[157]|SMRPM022-16|Mesostigmata|Macrochelidae|BOLD:ADA1133
Macrocheles merdarius|[158]|SMRPM023-16|Mesostigmata|Macrochelidae|BOLD:ADA1133
Macrocheles merdarius|[159]|SMRPM133-16|Mesostigmata|Macrochelidae|BOLD:ADA1133
Macrocheles merdarius|[160]|SMRPM485-17|Mesostigmata|Macrochelidae|BOLD:ADA1133
Macrocheles merdarius|[161]|SMRPM502-17|Mesostigmata|Macrochelidae
Macrocheles merdarius|[162]|SMRPM503-17|Mesostigmata|Macrochelidae|BOLD:ADA1540
Macrocheles merdarius|[163]|SMRPM504-17|Mesostigmata|Macrochelidae|BOLD:ADA1540
Macrocheles merdarius|[164]|SMRPM498-17|Mesostigmata|Macrochelidae|BOLD:ADA1540
Macrocheles merdarius|[165]|SMRPM436-16|Mesostigmata|Macrochelidae|BOLD:ADA1540
Macrocheles merdarius|[166]|SMRPM437-16|Mesostigmata|Macrochelidae|BOLD:ADA1540
Macrocheles merdarius|[167]|SMRPM497-17|Mesostigmata|Macrochelidae|BOLD:ADA1540
Macrocheles merdarius|[168]|SMRPM499-17|Mesostigmata|Macrochelidae|BOLD:ADA1540
Macrocheles merdarius|[169]|SMRPM509-17|Mesostigmata|Macrochelidae|BOLD:ADA1540
Macrocheles scutatiformis|[170]|SMRPM631-17|Mesostigmata|Macrochelidae|BOLD:AAH6540
Androlaelaps|[171]|SMRPM137-16|Mesostigmata|Laelapidae|BOLD:ADA1134
Androlaelaps casalis|[172]|SMRPM405-16|Mesostigmata|Laelapidae|BOLD:ADA2853
Androlaelaps casalis|[173]|SMRPM414-16|Mesostigmata|Laelapidae|BOLD:ADA2853
Androlaelaps casalis|[174]|SMRPM407-16|Mesostigmata|Laelapidae|BOLD:ADA2853
Androlaelaps casalis|[175]|SMRPM413-16|Mesostigmata|Laelapidae|BOLD:ADA2853
Androlaelaps casalis|[176]|SMRPM409-16|Mesostigmata|Laelapidae|BOLD:ADA2853
Androlaelaps casalis|[177]|SMRPM415-16|Mesostigmata|Laelapidae|BOLD:ADA2853
Androlaelaps casalis|[178]|SMRPM416-16|Mesostigmata|Laelapidae|BOLD:ADA2853
Androlaelaps casalis|[179]|SMRPM417-16|Mesostigmata|Laelapidae|BOLD:ADA2853
Androlaelaps casalis|[180]|SMRPM418-16|Mesostigmata|Laelapidae|BOLD:ADA2853
Gaeolaelaps aculeifer|[181]|SMRPM084-16|Mesostigmata|Laelapidae|BOLD:ABW8110
Gaeolaelaps aculeifer|[182]|SMRPM086-16|Mesostigmata|Laelapidae|BOLD:ABW8110
Gaeolaelaps aculeifer|[183]|SMRPM087-16|Mesostigmata|Laelapidae|BOLD:ABW8110
Gaeolaelaps aculeifer|[184]|SMRPM088-16|Mesostigmata|Laelapidae|BOLD:ABW8110
Gaeolaelaps aculeifer|[185]|SMRPM360-16|Mesostigmata|Laelapidae|BOLD:ABW8110
Laelapidae|[186]|SMRPM166-16|Mesostigmata|Laelapidae
Laelapidae|[187]|SMRPM172-16|Mesostigmata|Laelapidae
Ornithonyssus sylviarum|[188]|SMRPM012-16|Mesostigmata|Macronyssidae|BOLD:ADA5460
Ornithonyssus sylviarum|[189]|SMRPM013-16|Mesostigmata|Macronyssidae|BOLD:ADA5460
Ornithonyssus sylviarum|[190]|SMRPM011-16|Mesostigmata|Macronyssidae|BOLD:ADA5460
Ornithonyssus sylviarum|[191]|SMRPM014-16|Mesostigmata|Macronyssidae|BOLD:ADA5460
Ornithonyssus sylviarum|[192]|SMRPM015-16|Mesostigmata|Macronyssidae|BOLD:ADA5460
Rhodacarellus silesiacus|[193]|SMRPM176-16|Mesostigmata|Rhodacaridae|BOLD:ADA2698
Rhodacarellus silesiacus|[194]|SMRPM171-16|Mesostigmata|Rhodacaridae|BOLD:ADA1247
Rhodacarellus silesiacus|[195]|SMRPM177-16|Mesostigmata|Rhodacaridae|BOLD:ADA1247
Trematuridae|[196]|SMRPM658-17|Mesostigmata|Trematuridae|BOLD:ADG0003
Trichouropoda ovalis|[197]|SMRPM075-16|Mesostigmata|Trematuridae|BOLD:ADA4889
Trichouropoda ovalis|[198]|SMRPM074-16|Mesostigmata|Trematuridae|BOLD:ADA4889
Trichouropoda ovalis|[199]|SMRPM073-16|Mesostigmata|Trematuridae|BOLD:ADA4889
Trichouropoda ovalis|[200]|SMRPM076-16|Mesostigmata|Trematuridae|BOLD:ADA4889
Uroobovella marginata|[201]|SMRPM390-16|Mesostigmata|Urodinychidae|BOLD:ADA2195
Uroobovella fimicola|[202]|SMRPM385-16|Mesostigmata|Urodinychidae|BOLD:ACM5222
Uroobovella fimicola|[203]|SMRPM382-16|Mesostigmata|Urodinychidae|BOLD:ACM5222
Uroobovella fimicola|[204]|SMRPM383-16|Mesostigmata|Urodinychidae|BOLD:ACM5222
Uroobovella fimicola|[205]|SMRPM384-16|Mesostigmata|Urodinychidae|BOLD:ACM5222
Uroobovella fimicola|[206]|SMRPM452-16|Mesostigmata|Urodinychidae|BOLD:ACM5222
Uroobovella fimicola|[207]|SMRPM615-17|Mesostigmata|Urodinychidae|BOLD:ACM5222
Trichouropoda orbicularis|[208]|SMRPM161-16|Mesostigmata|Trematuridae|BOLD:ADA2194
Trichouropoda orbicularis|[209]|SMRPM312-16|Mesostigmata|Trematuridae|BOLD:ADA2194
Trichouropoda orbicularis|[210]|SMRPM313-16|Mesostigmata|Trematuridae|BOLD:ADA2194
Trichouropoda orbicularis|[211]|SMRPM314-16|Mesostigmata|Trematuridae|BOLD:ADA2194
Uropoda orbicularis|[212]|SMRPM315-16|Mesostigmata|Uropodidae|BOLD:ADA2406
Uropoda orbicularis|[213]|SMRPM316-16|Mesostigmata|Uropodidae|BOLD:ADA2406
Uropoda orbicularis|[214]|SMRPM318-16|Mesostigmata|Uropodidae|BOLD:ADA2406
Uropoda orbicularis|[215]|SMRPM319-16|Mesostigmata|Uropodidae|BOLD:ADA2406
Uroseius|[216]|SMRPM236-16|Mesostigmata|Polyaspididae|BOLD:ADB0621
Uroseius|[217]|SMRPM238-16|Mesostigmata|Polyaspididae|BOLD:ADB0621
Parasitus hyalinus|[218]|SMRPM662-17|Mesostigmata|Parasitidae|BOLD:ABW8099
Parasitus hyalinus|[219]|SMRPM091-16|Mesostigmata|Parasitidae|BOLD:ABW8099
Parasitus hyalinus|[220]|SMRPM092-16|Mesostigmata|Parasitidae|BOLD:ABW8099
Parasitus hyalinus|[221]|SMRPM089-16|Mesostigmata|Parasitidae|BOLD:ABW8099
Parasitus hyalinus|[222]|SMRPM090-16|Mesostigmata|Parasitidae|BOLD:ABW8099
Parasitus hyalinus|[223]|SMRPM093-16|Mesostigmata|Parasitidae|BOLD:ABW8099
Parasitus hyalinus|[224]|SMRPM548-17|Mesostigmata|Parasitidae|BOLD:ABW8099
Parasitus hyalinus|[225]|SMRPM549-17|Mesostigmata|Parasitidae|BOLD:ABW8099
Parasitus hyalinus|[226]|SMRPM659-17|Mesostigmata|Parasitidae|BOLD:ABW8099
Parasitus hyalinus|[227]|SMRPM660-17|Mesostigmata|Parasitidae|BOLD:ABW8099
Parasitus hyalinus|[228]|SMRPM661-17|Mesostigmata|Parasitidae|BOLD:ABW8099
Parasitus hyalinus|[229]|SMRPM663-17|Mesostigmata|Parasitidae|BOLD:ABW8099

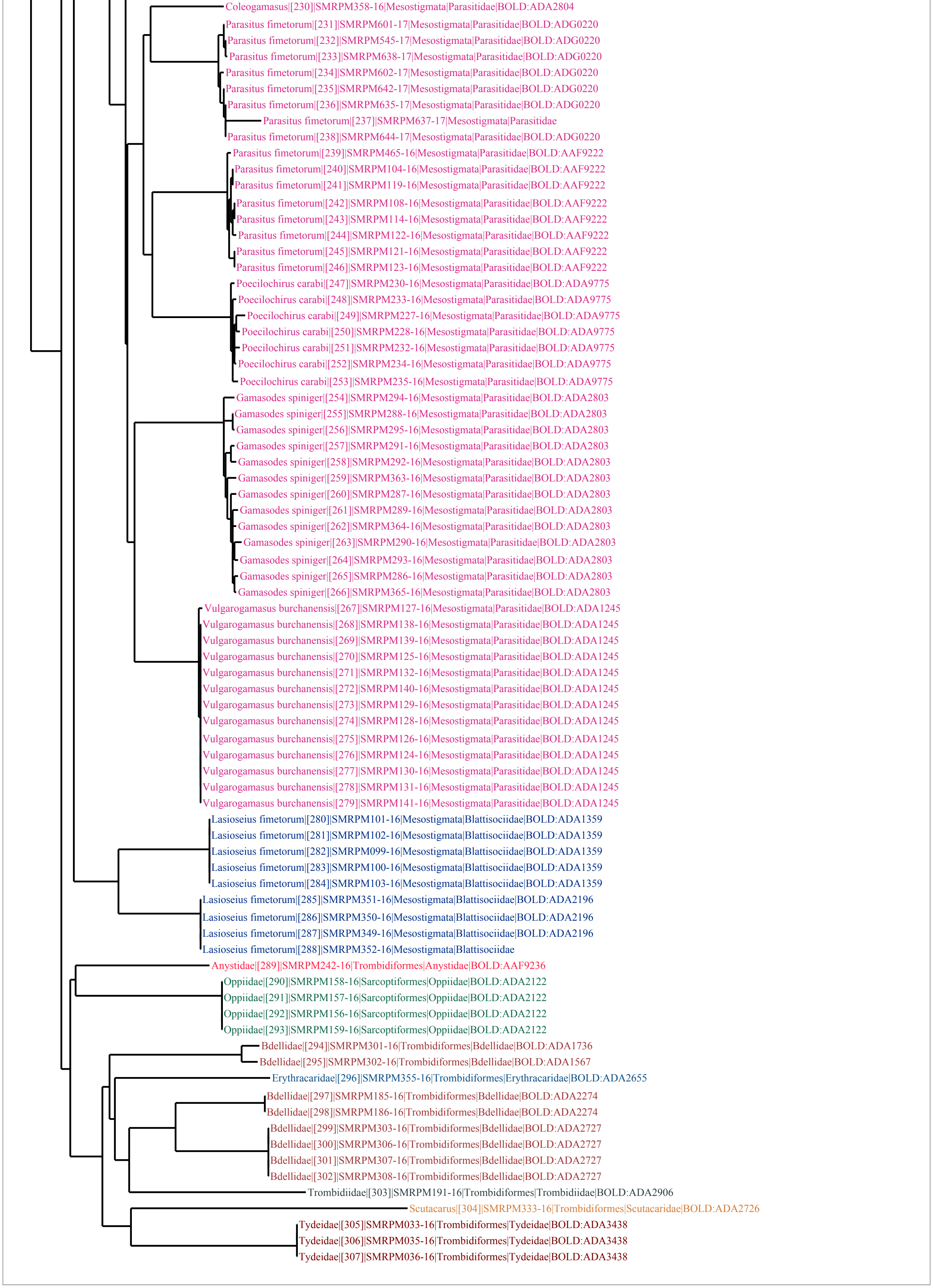
Coleogamasus|[230]|SMRPM358-16|Mesostigmata|Parasitidae|BOLD:ADA2804
Parasitus fimetorum|[231]|SMRPM601-17|Mesostigmata|Parasitidae|BOLD:ADG0220
Parasitus fimetorum|[232]|SMRPM545-17|Mesostigmata|Parasitidae|BOLD:ADG0220
Parasitus fimetorum|[233]|SMRPM638-17|Mesostigmata|Parasitidae|BOLD:ADG0220
Parasitus fimetorum|[234]|SMRPM602-17|Mesostigmata|Parasitidae|BOLD:ADG0220
Parasitus fimetorum|[235]|SMRPM642-17|Mesostigmata|Parasitidae|BOLD:ADG0220
Parasitus fimetorum|[236]|SMRPM635-17|Mesostigmata|Parasitidae|BOLD:ADG0220
Parasitus fimetorum|[237]|SMRPM637-17|Mesostigmata|Parasitidae
Parasitus fimetorum|[238]|SMRPM644-17|Mesostigmata|Parasitidae|BOLD:ADG0220
Parasitus fimetorum|[239]|SMRPM465-16|Mesostigmata|Parasitidae|BOLD:AAF9222
Parasitus fimetorum|[240]|SMRPM104-16|Mesostigmata|Parasitidae|BOLD:AAF9222
Parasitus fimetorum|[241]|SMRPM119-16|Mesostigmata|Parasitidae|BOLD:AAF9222
Parasitus fimetorum|[242]|SMRPM108-16|Mesostigmata|Parasitidae|BOLD:AAF9222
Parasitus fimetorum|[243]|SMRPM114-16|Mesostigmata|Parasitidae|BOLD:AAF9222
Parasitus fimetorum|[244]|SMRPM122-16|Mesostigmata|Parasitidae|BOLD:AAF9222
Parasitus fimetorum|[245]|SMRPM121-16|Mesostigmata|Parasitidae|BOLD:AAF9222
Parasitus fimetorum|[246]|SMRPM123-16|Mesostigmata|Parasitidae|BOLD:AAF9222
Poecilochirus carabi|[247]|SMRPM230-16|Mesostigmata|Parasitidae|BOLD:ADA9775
Poecilochirus carabi|[248]|SMRPM233-16|Mesostigmata|Parasitidae|BOLD:ADA9775
Poecilochirus carabi|[249]|SMRPM227-16|Mesostigmata|Parasitidae|BOLD:ADA9775
Poecilochirus carabi|[250]|SMRPM228-16|Mesostigmata|Parasitidae|BOLD:ADA9775
Poecilochirus carabi|[251]|SMRPM232-16|Mesostigmata|Parasitidae|BOLD:ADA9775
Poecilochirus carabi|[252]|SMRPM234-16|Mesostigmata|Parasitidae|BOLD:ADA9775
Poecilochirus carabi|[253]|SMRPM235-16|Mesostigmata|Parasitidae|BOLD:ADA9775
Gamasodes spiniger|[254]|SMRPM294-16|Mesostigmata|Parasitidae|BOLD:ADA2803
Gamasodes spiniger|[255]|SMRPM288-16|Mesostigmata|Parasitidae|BOLD:ADA2803
Gamasodes spiniger|[256]|SMRPM295-16|Mesostigmata|Parasitidae|BOLD:ADA2803
Gamasodes spiniger|[257]|SMRPM291-16|Mesostigmata|Parasitidae|BOLD:ADA2803
Gamasodes spiniger|[258]|SMRPM292-16|Mesostigmata|Parasitidae|BOLD:ADA2803
Gamasodes spiniger|[259]|SMRPM363-16|Mesostigmata|Parasitidae|BOLD:ADA2803
Gamasodes spiniger|[260]|SMRPM287-16|Mesostigmata|Parasitidae|BOLD:ADA2803
Gamasodes spiniger|[261]|SMRPM289-16|Mesostigmata|Parasitidae|BOLD:ADA2803
Gamasodes spiniger|[262]|SMRPM364-16|Mesostigmata|Parasitidae|BOLD:ADA2803
Gamasodes spiniger|[263]|SMRPM290-16|Mesostigmata|Parasitidae|BOLD:ADA2803
Gamasodes spiniger|[264]|SMRPM293-16|Mesostigmata|Parasitidae|BOLD:ADA2803
Gamasodes spiniger|[265]|SMRPM286-16|Mesostigmata|Parasitidae|BOLD:ADA2803
Gamasodes spiniger|[266]|SMRPM365-16|Mesostigmata|Parasitidae|BOLD:ADA2803
Vulgarogamasus burchanensis|[267]|SMRPM127-16|Mesostigmata|Parasitidae|BOLD:ADA1245
Vulgarogamasus burchanensis|[268]|SMRPM138-16|Mesostigmata|Parasitidae|BOLD:ADA1245
Vulgarogamasus burchanensis|[269]|SMRPM139-16|Mesostigmata|Parasitidae|BOLD:ADA1245
Vulgarogamasus burchanensis|[270]|SMRPM125-16|Mesostigmata|Parasitidae|BOLD:ADA1245
Vulgarogamasus burchanensis|[271]|SMRPM132-16|Mesostigmata|Parasitidae|BOLD:ADA1245
Vulgarogamasus burchanensis|[272]|SMRPM140-16|Mesostigmata|Parasitidae|BOLD:ADA1245
Vulgarogamasus burchanensis|[273]|SMRPM129-16|Mesostigmata|Parasitidae|BOLD:ADA1245
Vulgarogamasus burchanensis|[274]|SMRPM128-16|Mesostigmata|Parasitidae|BOLD:ADA1245
Vulgarogamasus burchanensis|[275]|SMRPM126-16|Mesostigmata|Parasitidae|BOLD:ADA1245
Vulgarogamasus burchanensis|[276]|SMRPM124-16|Mesostigmata|Parasitidae|BOLD:ADA1245
Vulgarogamasus burchanensis|[277]|SMRPM130-16|Mesostigmata|Parasitidae|BOLD:ADA1245
Vulgarogamasus burchanensis|[278]|SMRPM131-16|Mesostigmata|Parasitidae|BOLD:ADA1245
Vulgarogamasus burchanensis|[279]|SMRPM141-16|Mesostigmata|Parasitidae|BOLD:ADA1245
Lasioseius fimetorum|[280]|SMRPM101-16|Mesostigmata|Blattisociidae|BOLD:ADA1359
Lasioseius fimetorum|[281]|SMRPM102-16|Mesostigmata|Blattisociidae|BOLD:ADA1359
Lasioseius fimetorum|[282]|SMRPM099-16|Mesostigmata|Blattisociidae|BOLD:ADA1359
Lasioseius fimetorum|[283]|SMRPM100-16|Mesostigmata|Blattisociidae|BOLD:ADA1359
Lasioseius fimetorum|[284]|SMRPM103-16|Mesostigmata|Blattisociidae|BOLD:ADA1359
Lasioseius fimetorum|[285]|SMRPM351-16|Mesostigmata|Blattisociidae|BOLD:ADA2196
Lasioseius fimetorum|[286]|SMRPM350-16|Mesostigmata|Blattisociidae|BOLD:ADA2196
Lasioseius fimetorum|[287]|SMRPM349-16|Mesostigmata|Blattisociidae|BOLD:ADA2196
Lasioseius fimetorum|[288]|SMRPM352-16|Mesostigmata|Blattisociidae
Anystidae|[289]|SMRPM242-16|Trombidiformes|Anystidae|BOLD:AAF9236
Oppiidae|[290]|SMRPM158-16|Sarcoptiformes|Oppiidae|BOLD:ADA2122
Oppiidae|[291]|SMRPM157-16|Sarcoptiformes|Oppiidae|BOLD:ADA2122
Oppiidae|[292]|SMRPM156-16|Sarcoptiformes|Oppiidae|BOLD:ADA2122
Oppiidae|[293]|SMRPM159-16|Sarcoptiformes|Oppiidae|BOLD:ADA2122
Bdellidae|[294]|SMRPM301-16|Trombidiformes|Bdellidae|BOLD:ADA1736
Bdellidae|[295]|SMRPM302-16|Trombidiformes|Bdellidae|BOLD:ADA1567
Erythracaridae|[296]|SMRPM355-16|Trombidiformes|Erythracaridae|BOLD:ADA2655
Bdellidae|[297]|SMRPM185-16|Trombidiformes|Bdellidae|BOLD:ADA2274
Bdellidae|[298]|SMRPM186-16|Trombidiformes|Bdellidae|BOLD:ADA2274
Bdellidae|[299]|SMRPM303-16|Trombidiformes|Bdellidae|BOLD:ADA2727
Bdellidae|[300]|SMRPM306-16|Trombidiformes|Bdellidae|BOLD:ADA2727
Bdellidae|[301]|SMRPM307-16|Trombidiformes|Bdellidae|BOLD:ADA2727
Bdellidae|[302]|SMRPM308-16|Trombidiformes|Bdellidae|BOLD:ADA2727
Trombidiidae|[303]|SMRPM191-16|Trombidiformes|Trombidiidae|BOLD:ADA2906
Scutacarus|[304]|SMRPM333-16|Trombidiformes|Scutacaridae|BOLD:ADA2726
Tydeidae|[305]|SMRPM033-16|Trombidiformes|Tydeidae|BOLD:ADA3438
Tydeidae|[306]|SMRPM035-16|Trombidiformes|Tydeidae|BOLD:ADA3438
Tydeidae|[307]|SMRPM036-16|Trombidiformes|Tydeidae|BOLD:ADA3438

Supplementary Figure 2: Library of specimen images corresponding to taxon ID tree for all barcode compliant mite sequences.

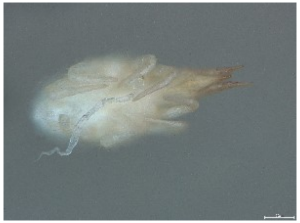

[1] HUJINVACA17 [Ventral]
Cunaxidae
BIN URI: BOLD:ADA5281

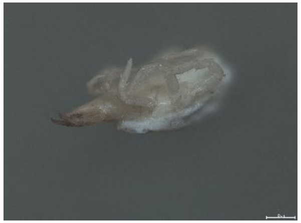

[2] HUJINVACA18 [Ventral]
Cunaxidae
BIN URI: BOLD:ADA5281

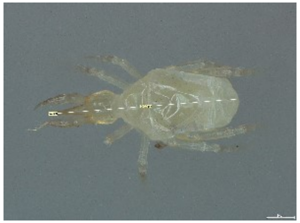

[3] HUJINVACA16 [Dorsal]
Cunaxidae
BIN URI: BOLD:ADA3701

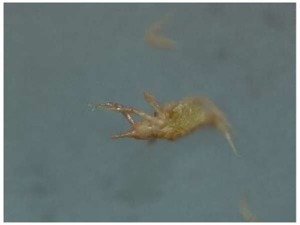

[4] HUJINVACA221 [Ventral]
Cunaxidae
BIN URI: BOLD:ADA2956

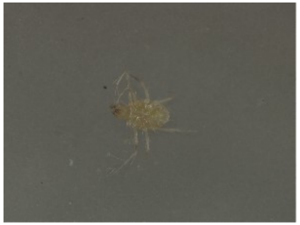

[5] HUJINVACA272 [Ventral]
Cheletomorpha lepidopterorum
BIN URI: BOLD:ADA2473

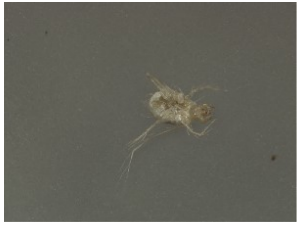

[6] HUJINVACA273 [Ventral]
Cheletomorpha lepidopterorum
BIN URI: BOLD:ADA2473

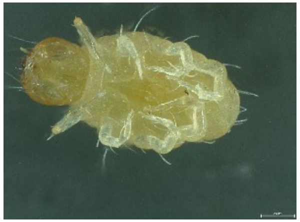

[7] HUJINVACA55 [Ventral]
Cheyletus bidentatus
BIN URI: BOLD:ACB3980

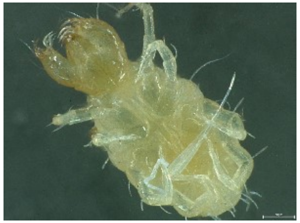

[8] HUJINVACA54 [Ventral]
Cheyletus bidentatus
BIN URI: BOLD:ACB3980

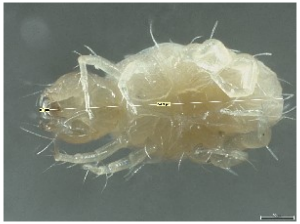

[9] HUJINVACA53 [Ventral]
Cheyletus bidentatus
BIN URI: BOLD:ACB3980

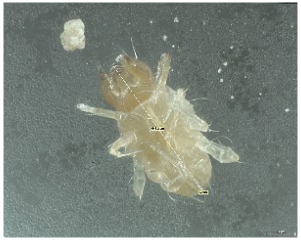

[10] HUJINVACA52 [Ventral]
Cheyletus bidentatus
BIN URI: BOLD:ACB3980

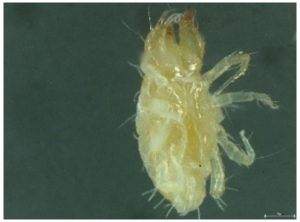

[11] HUJINVACA56 [Ventral]
Cheyletus bidentatus
BIN URI: BOLD:ACB3980

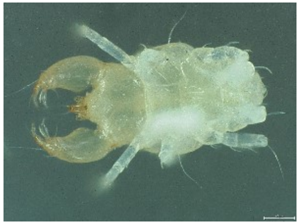

[12] HUJINVACA62 [Ventral]
Cheyletus bidentatus
BIN URI: BOLD:ACB3980

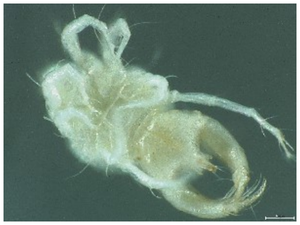

[13] HUJINVACA63 [Ventral]
Cheyletus bidentatus
BIN URI: BOLD:ACB3980

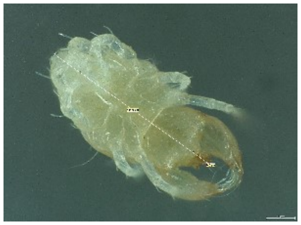

[14] HUJINVACA64 [Ventral]
Cheyletus bidentatus
BIN URI: BOLD:ACB3980

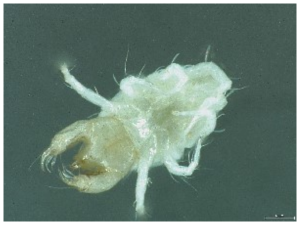

[15] HUJINVACA65 [Ventral]
Cheyletus bidentatus
BIN URI: BOLD:ACB3980

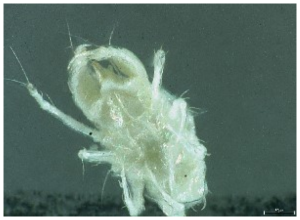

[16] HUJINVACA66 [Ventral]
Cheyletus bidentatus
BIN URI: BOLD:ACB3980

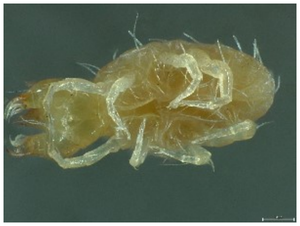

[17] HUJINVACA49 [Ventral]
Cheyletus bidentatus
BIN URI: BOLD:ACB3980

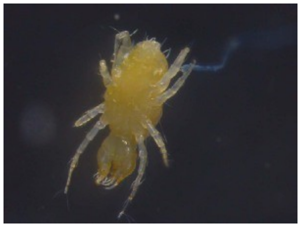

[18] MZNA442883 [Ventral]
Cheyletus bidentatus
BIN URI: BOLD:ACB3980

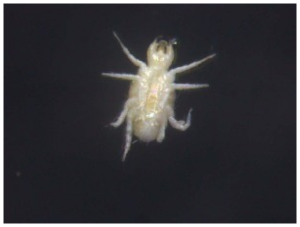

[19] MZNA442884 [Ventral]
Cheyletus bidentatus
BIN URI: BOLD:ACB3980

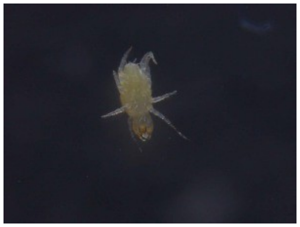

[20] MZNA442891 [Ventral]
Cheyletus bidentatus
BIN URI: BOLD:ACB3980

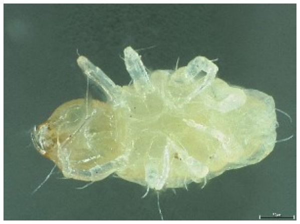

[21] HUJINVACA48 [Ventral]
Cheyletus malaccensis
BIN URI: BOLD:ACH5819

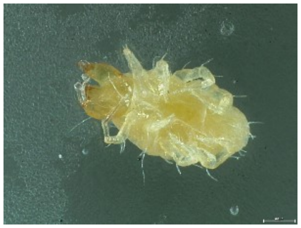

[22] HUJINVACA47 [Ventral]
Cheyletus malaccensis
BIN URI: BOLD:ACH5819

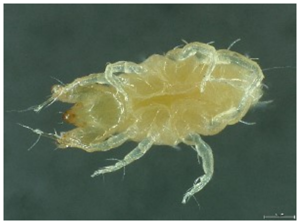

[23] HUJINVACA50 [Ventral]
Cheyletus malaccensis
BIN URI: BOLD:ACH5819

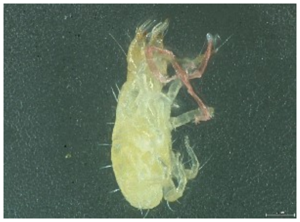

[24] HUJINVACA51 [Lateral]
Cheyletus malaccensis
BIN URI: BOLD:ACH5819

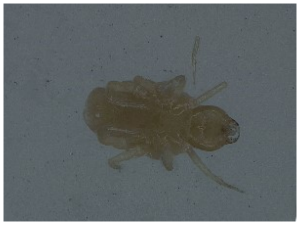

[25] HUJINVACA148 [Ventral]
Cheyletus malaccensis
BIN URI: BOLD:ACH5819

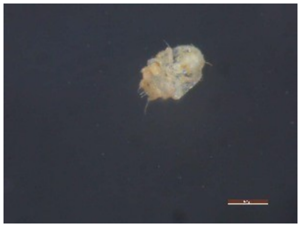

[26] MZNA442801 [Ventral]
Tetranychus urticae
BIN URI: BOLD:AAB9986

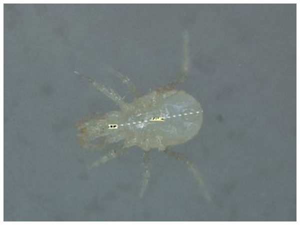

[27] HUJINVACA179 [Ventral]
Proctolaelaps
BIN URI: BOLD:ADA1249

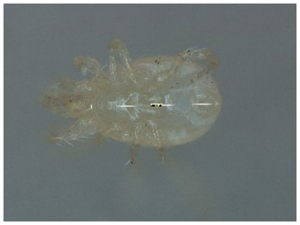

[28] HUJINVACA180 [Ventral]
Proctolaelaps
BIN URI: BOLD:ADA1249

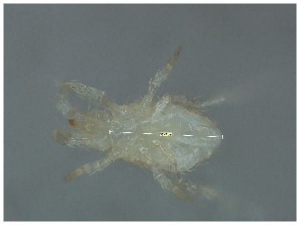

[29] HUJINVACA181 [Ventral]
Proctolaelaps
BIN URI: BOLD:ADA1249

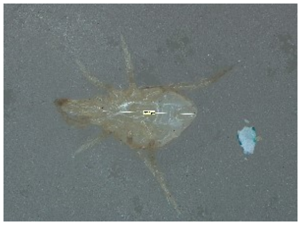

[30] HUJINVACA182 [Ventral]
Proctolaelaps
BIN URI: BOLD:ADA1249

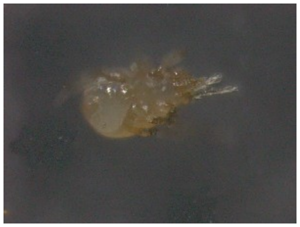

[31] HUJINVACA494 [Ventral]
Mesostigmata
BIN URI: BOLD:ADG1141

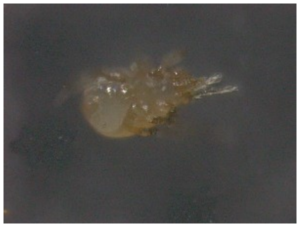

[32] HUJINVACA495 [Ventral]
Mesostigmata
BIN URI: BOLD:ADG1141

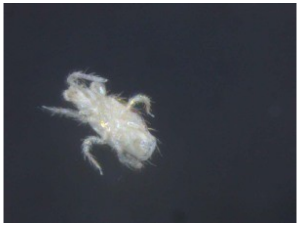

[33] MZNA442808 [Ventral]
Proctolaelaps parascolyti
BIN URI: BOLD:ADG2096

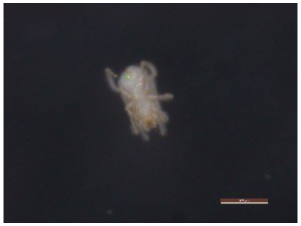

[34] MZNA442784 [Ventral]
Proctolaelaps pygmaeus
BIN URI: BOLD:ADG3898

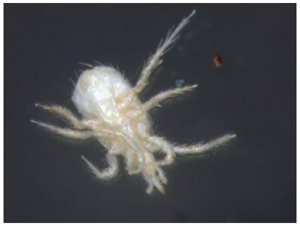

[35] MZNA442806 [Ventral]
Proctolaelaps pygmaeus
BIN URI: BOLD:ADG3898

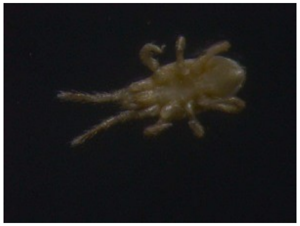

[36] MZNA442843 [Ventral]
Proctolaelaps pygmaeus
BIN URI: BOLD:ADG0088

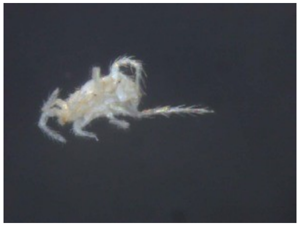

[37] MZNA442807 [Ventral]
Proctolaelaps pygmaeus
BIN URI: BOLD:ADG0088

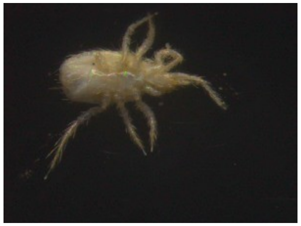

[38] MZNA442842 [Ventral]
Proctolaelaps parascolyti
BIN URI: BOLD:ADG0088

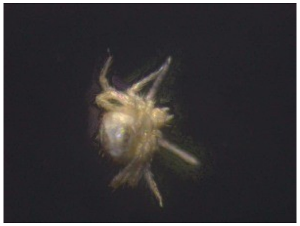

[39] MZNA442840 [Ventral]
Proctolaelaps pygmaeus
BIN URI: BOLD:ADG0088

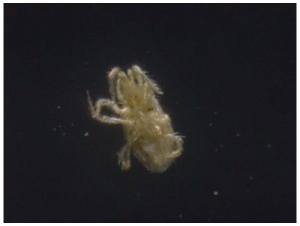

[40] MZNA442846 [Ventral]
Proctolaelaps pygmaeus
BIN URI: BOLD:ADG0088

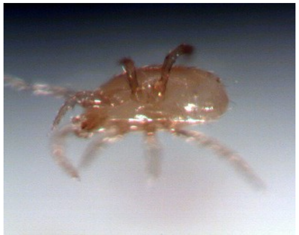

[41] POD0ME403 [Ventral]
Proctolaelaps scolyti
BIN URI: BOLD:ADA1147

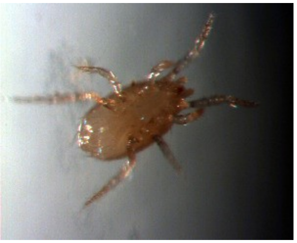

[42] POD0ME404 [Ventral]
Proctolaelaps scolyti
BIN URI: BOLD:ADA1147

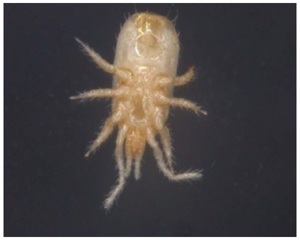

[43] MZNA442813 [Ventral]
Proctolaelaps parascolyti
BIN URI: BOLD:ADF9931

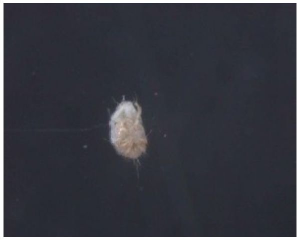

[44] MZNA442824 [Ventral]
Proctolaelaps parascolyti
BIN URI: BOLD:ADF9931

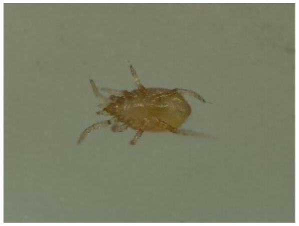

[45] HUJINVACA284 [Ventral]
Proctolaelaps parascolyti
BIN URI: BOLD:ADA2305

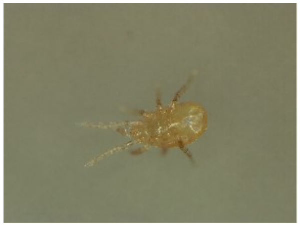

[46] HUJINVACA285 [Ventral]
Proctolaelaps parascolyti
BIN URI: BOLD:ADA2305

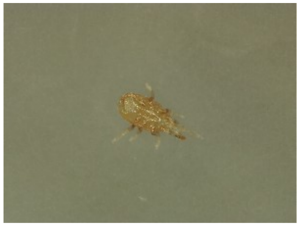

[47] HUJINVACA286 [Ventral]
Proctolaelaps parascolyti
BIN URI: BOLD:ADA2305

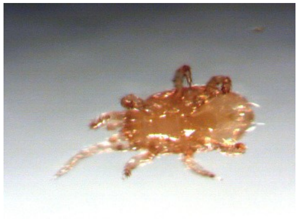

[48] PAP1ME401 [Ventral]
Proctolaelaps scolyti
BIN URI: BOLD:ADA1466

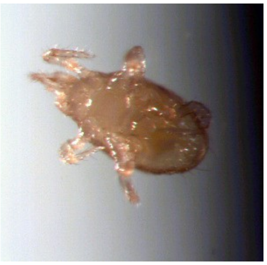

[49] PAP1ME402 [Ventral]
Proctolaelaps scolyti
BIN URI: BOLD:ADA1466

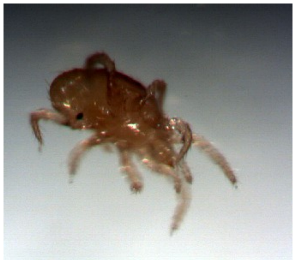

[50] PAP1ME410 [Ventral]
Proctolaelaps scolyti
BIN URI: BOLD:ADA1466

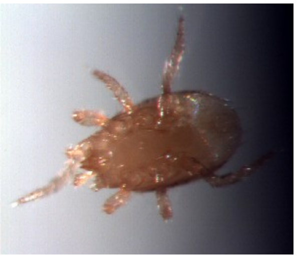

[51] PAP1ME405 [Ventral]
Proctolaelaps scolyti
BIN URI: BOLD:ADA1466

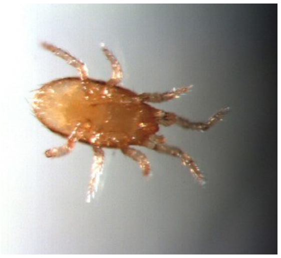

[52] PAP1ME407 [Ventral]
Proctolaelaps scolyti
BIN URI: BOLD:ADA1466

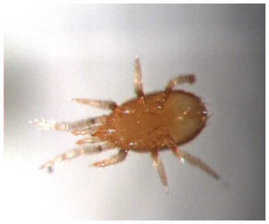

[53] PAP1ME411 [Ventral]
Proctolaelaps scolyti
BIN URI: BOLD:ADA1466

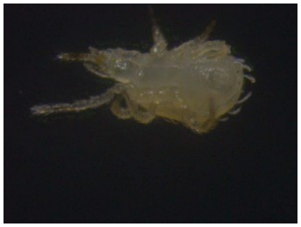

[54] MZNA442844 [Ventral]
Ameroseius
BIN URI: BOLD:ADF9704

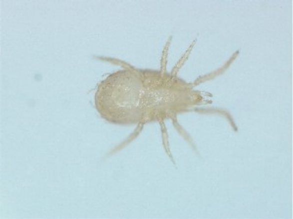

[55] HUJINVACA208 [Ventral]
Ameroseius eumorphus
BIN URI: BOLD:ADA2405

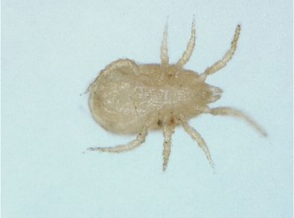

[56] HUJINVACA207 [Ventral]
Ameroseius eumorphus
BIN URI: BOLD:ADA2405

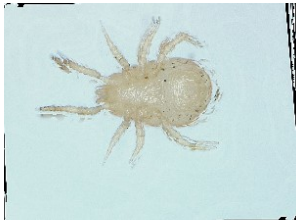

[57] HUJINVACA209 [Ventral]
Ameroseius eumorphus
BIN URI: BOLD:ADA2405

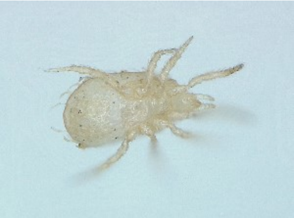

[58] HUJINVACA210 [Ventral]
Ameroseius eumorphus
BIN URI: BOLD:ADA2405

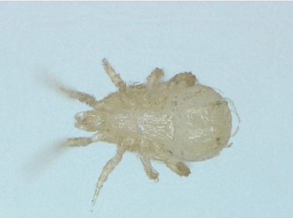

[59] HUJINVACA211 [Ventral]
Ameroseius eumorphus
BIN URI: BOLD:ADA2405

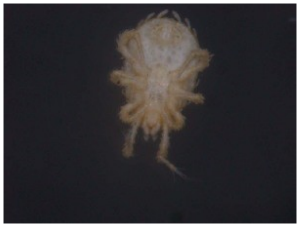

[60] MZNA442778 [Ventral]
Ameroseius macrochelae
BIN URI: BOLD:ADG0422

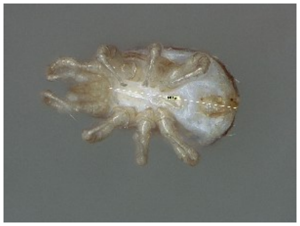

[61] HUJINVACA136 [Ventral]
Ascidae
BIN URI: BOLD:ACT7095

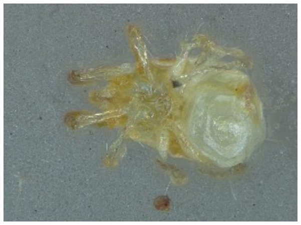

[62] HUJINVACA178 [Ventral]
Phytoseiidae
BIN URI: BOLD:ACO6087

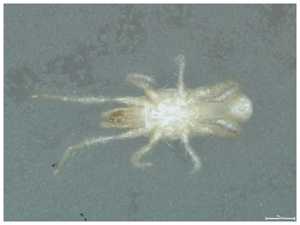

[63] HUJINVACA24 [Ventral]
Protogamasellopsis corticalis
BIN URI: BOLD:ADA3054

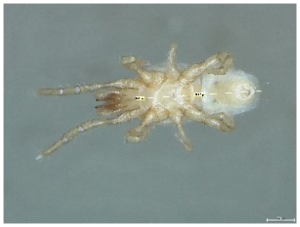

[64] HUJINVACA25 [Ventral]
Protogamasellopsis corticalis
BIN URI: BOLD:ADA3054

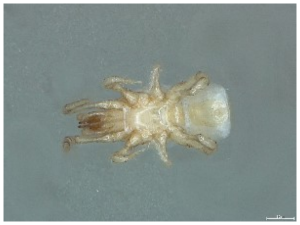

[65] HUJINVACA26 [Ventral]
Protogamasellopsis corticalis
BIN URI: BOLD:ADA3054

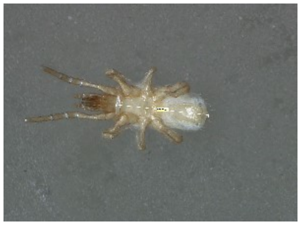

[66] HUJINVACA135 [Ventral]
Protogamasellopsis corticalis
BIN URI: BOLD:ADA3054

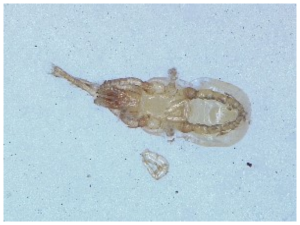

[67] HUJINVACA95 [Ventral]
Protogamasellopsis corticalis
BIN URI: BOLD:ADA1246

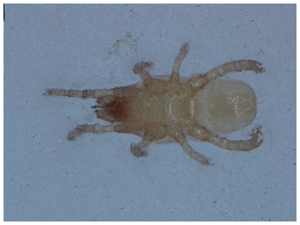

[68] HUJINVACA97 [Ventral]
Protogamasellopsis corticalis
BIN URI: BOLD:ADA1246

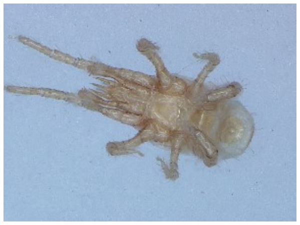

[69] HUJINVACA98 [Ventral]
Protogamasellopsis corticalis
BIN URI: BOLD:ADA1246

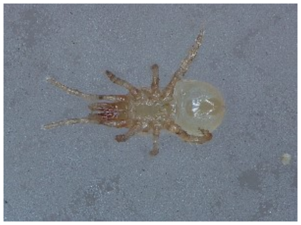

[70] HUJINVACA94 [Ventral]
Protogamasellopsis corticalis
BIN URI: BOLD:ADA1246

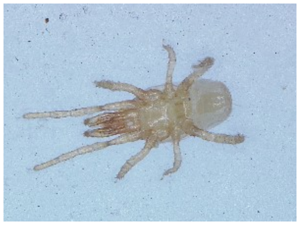

[71] HUJINVACA96 [Ventral]
Protogamasellopsis corticalis
BIN URI: BOLD:ADA1246

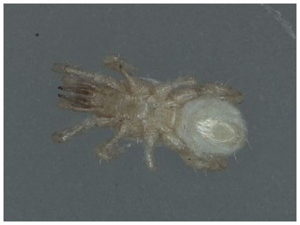

[72] HUJINVACA175 [Ventral]
Protogamasellopsis corticalis
BIN URI: BOLD:ADA1246

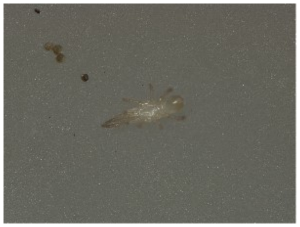

[73] HUJINVACA268 [Ventral]
Protogamasellopsis corticalis
BIN URI: BOLD:ADA2499

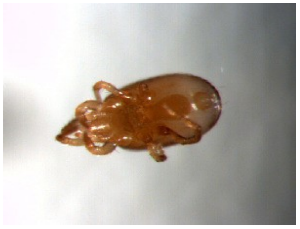

[74] CON6ME202 [Ventral]
Androlaelaps casalis
BIN URI: BOLD:ADA1701

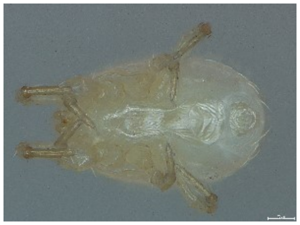

[75] HUJINVACA8 [Ventral]
Androlaelaps casalis
BIN URI: BOLD:ADA1701

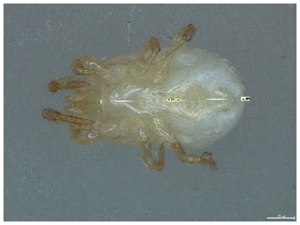

[76] HUJINVACA9 [Ventral]
Androlaelaps casalis
BIN URI: BOLD:ADA1701

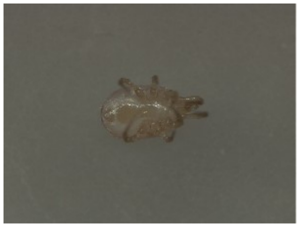

[77] HUJINVACA278 [Ventral]
Androlaelaps casalis

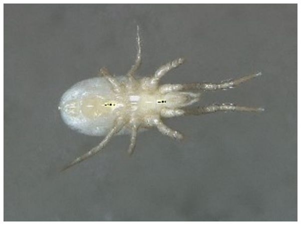

[78] HUJINVACA134 [Ventral]
Laelapidae
BIN URI: BOLD:ADA3055

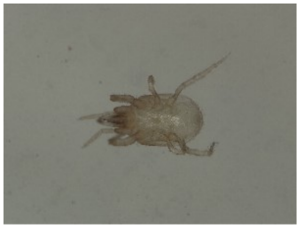

[79] HUJINVACA279 [Ventral]
Stratiolaelaps scimitus
BIN URI: BOLD:AAF3914

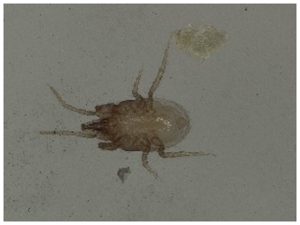

[80] HUJINVACA280 [Ventral]
Stratiolaelaps scimitus
BIN URI: BOLD:AAF3914

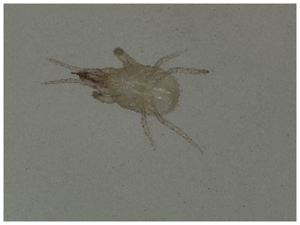

[81] HUJINVACA282 [Ventral]
Stratiolaelaps scimitus
BIN URI: BOLD:AAF3914

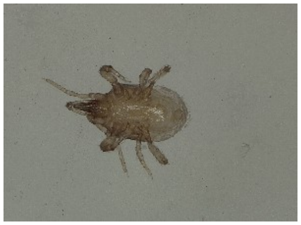

[82] HUJINVACA283 [Ventral]
Stratiolaelaps scimitus
BIN URI: BOLD:AAF3914

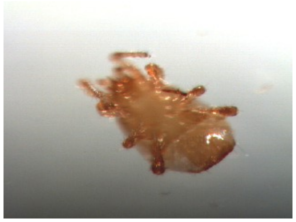

[83] BER2ME114 [Ventral]
Dendrolaealps presepum
BIN URI: BOLD:ADA1612

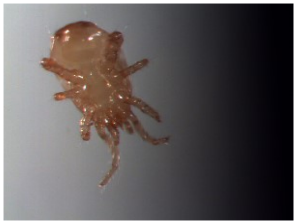

[84] BER2ME112 [Ventral]
Dendrolaealps presepum
BIN URI: BOLD:ADA1612

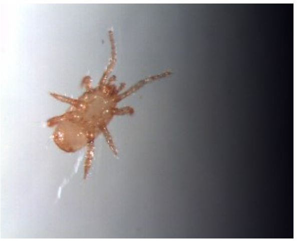

[85] BER2ME111 [Ventral]
Dendrolaealps presepum
BIN URI: BOLD:ADA1612

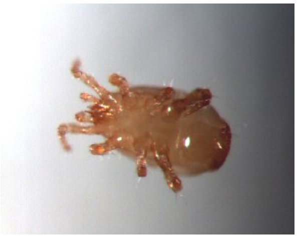

[86] BER2ME113 [Ventral]
Dendrolaealps presepum
BIN URI: BOLD:ADA1612

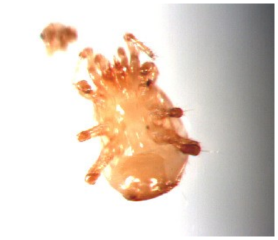

[87] PAP1ME121 [Ventral]
Dendrolaealps presepum
BIN URI: BOLD:ADA1612

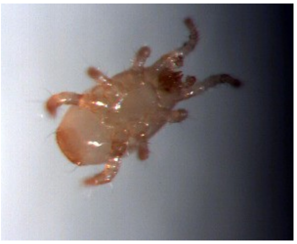

[88] PAP1ME122 [Ventral]
Dendrolaealps presepum
BIN URI: BOLD:ADA1612

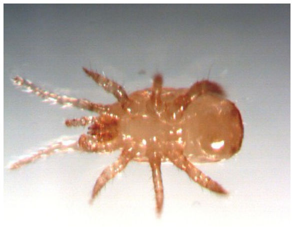

[89] PAP1ME123 [Ventral]
Dendrolaealps presepum
BIN URI: BOLD:ADA1612

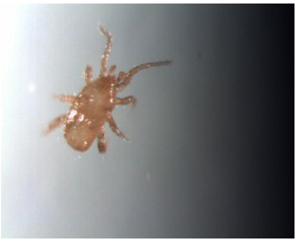

[90] RIG1ME108 [Dorsal]
Dendrolaealps presepum
BIN URI: BOLD:ACW5179

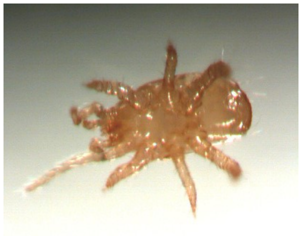

[91] PAP1ME124 [Ventral]
Dendrolaealps presepum
BIN URI: BOLD:ACW5179

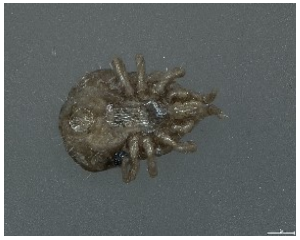

[92] UA-NSTX290 [Ventral]
Dermanyssus carpathicus
BIN URI: BOLD:ADA1047

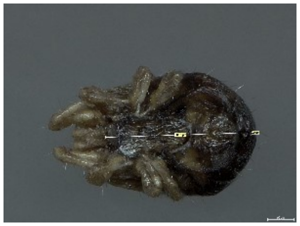

[93] UA-NSTX295 [Ventral]
Dermanyssus carpathicus
BIN URI: BOLD:ADA1047

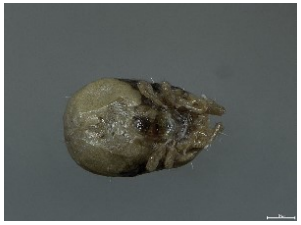

[94] UA-NSTX291 [Ventral]
Dermanyssus carpathicus
BIN URI: BOLD:ADA1047

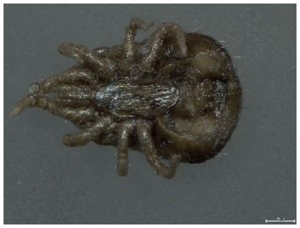

[95] UA-NSTX293 [Ventral]
Dermanyssus carpathicus
BIN URI: BOLD:ADA1047

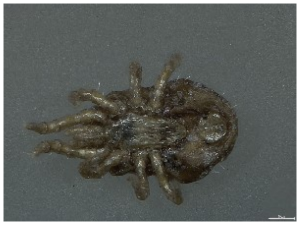

[96] UA-NSTX-264 [Ventral]
Dermanyssus carpathicus
BIN URI: BOLD:ADA1047

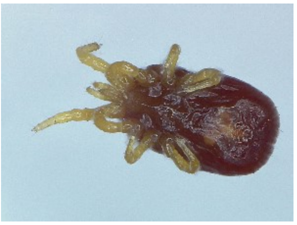

[97] HUJINVACA194 [Ventral]
Dermanyssus gallinae
BIN URI: BOLD:AAC4428

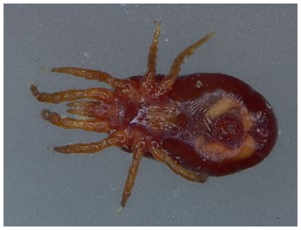

[98] HUJINVACA4 [Ventral]
Dermanyssus gallinae
BIN URI: BOLD:AAC4428

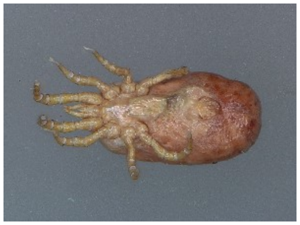

[99] HUJINVACA3 [Ventral]
Dermanyssus gallinae
BIN URI: BOLD:AAC4428

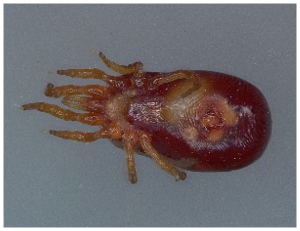

[100] HUJINVACA5 [Ventral]
Dermanyssus gallinae
BIN URI: BOLD:AAC4428

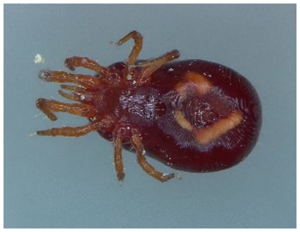

[101] HUJINVACA2 [Ventral]
Dermanyssus gallinae
BIN URI: BOLD:AAC4428

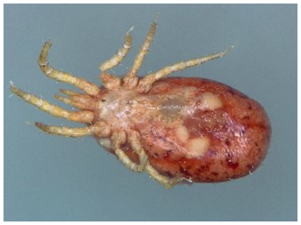

[102] HUJINVACA1 [Ventral]
Dermanyssus gallinae
BIN URI: BOLD:AAC4428

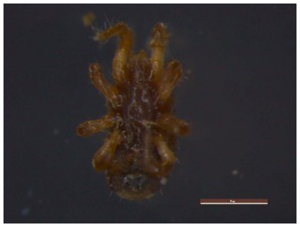

[103] MZNA442892 [Ventral]
Dermanyssus gallinae
BIN URI: BOLD:AAC4428

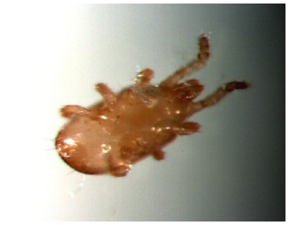

[104] POK0ME115 [Ventral]
Dendrolaelaps presepum
BIN URI: BOLD:ADA1360

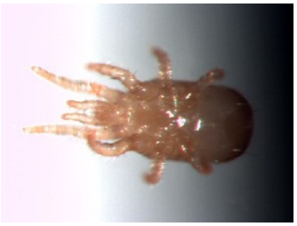

[105] POK0ME117 [Ventral]
Dendrolaealaps presepum
BIN URI: BOLD:ADA1360

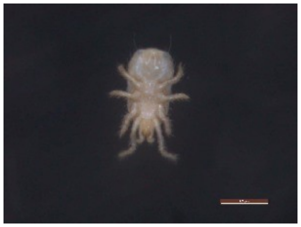

[106] MZNA442783 [Ventral]
Dendrolaealps longiscutatus
BIN URI: BOLD:ADA1360

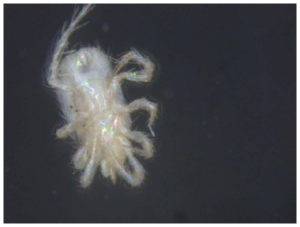

[107] MZNA442809 [Ventral]
Dendrolaealps longiscutatus
BIN URI: BOLD:ADA1360

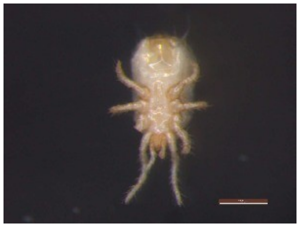

[108] MZNA442751 [Ventral]
Dendrolaealps longiscutatus
BIN URI: BOLD:ADA1360

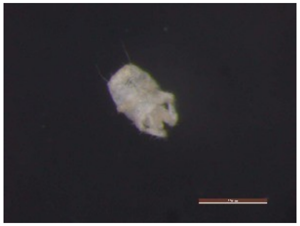

[109] MZNA442757 [Ventral]
Dendrolaealps longiscutatus
BIN URI: BOLD:ADA1360

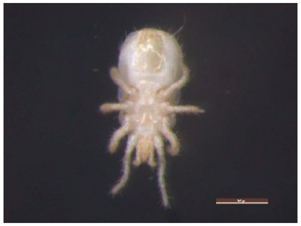

[110] MZNA442756 [Ventral]
Dendrolaealps longiscutatus
BIN URI: BOLD:ADA1360

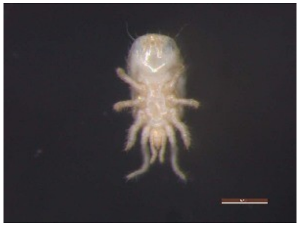

[111] MZNA442747 [Ventral]
Dendrolaealps longiscutatus
BIN URI: BOLD:ADA1360

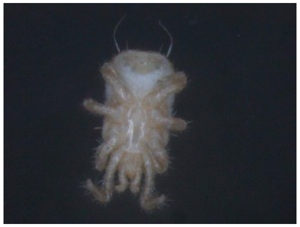

[112] MZNA442794 [Ventral]
Dendrolaealps longiscutatus
BIN URI: BOLD:ADA1360

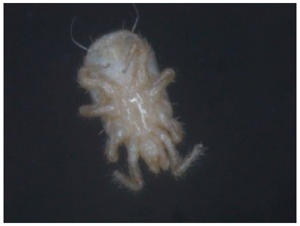

[113] MZNA442795 [Ventral]
Dendrolaealps longiscutatus
BIN URI: BOLD:ADA1360

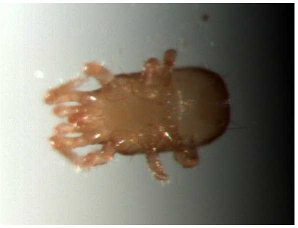

[114] POK0ME116 [Ventral]
Dendrolaealps presepum
BIN URI: BOLD:ADA1360

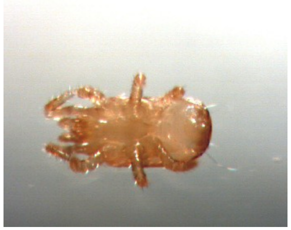

[115] GER3ME119 [Ventral]
Dendrolaealps presepum
BIN URI: BOLD:ADA1360

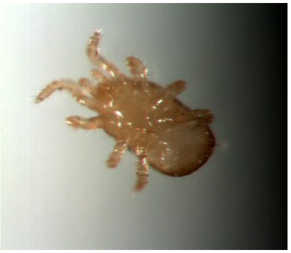

[116] GER3ME120 [Ventral]
Dendrolaealps presepum
BIN URI: BOLD:ADA1360

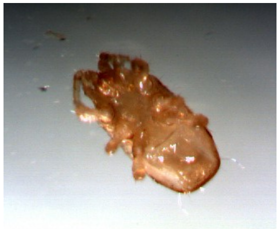

[117] GER3ME121 [Ventral]
Dendrolaealps presepum
BIN URI: BOLD:ADA1360

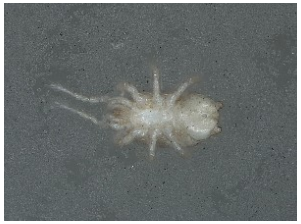

[118] HUJINVACA170 [Ventral]
Dendrolaelaps lobatus

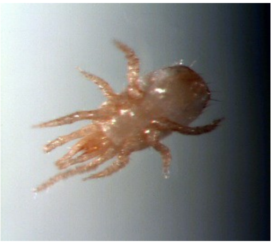

[119] GER3ME101 [Ventral]
Digamasellidae
BIN URI: BOLD:ADA1362

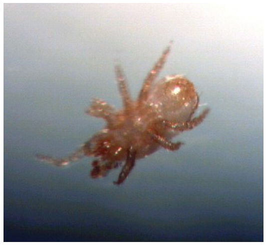

[120] GER3ME102 [Ventral]
Digamasellidae
BIN URI: BOLD:ADA1362

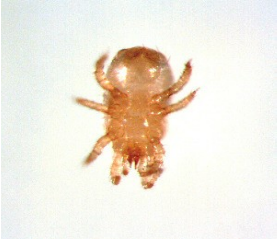

[121] GER3ME103 [Ventral]
Digamasellidae
BIN URI: BOLD:ADA1362

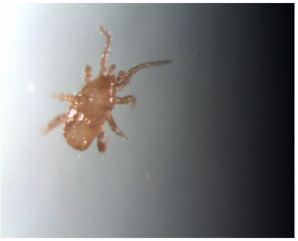

[122] RIG1ME107 [Dorsal]
Digamasellidae
BIN URI: BOLD:ADA1362

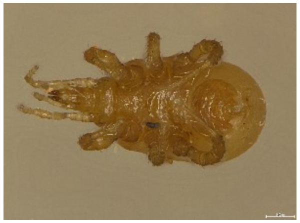

[123] UA-NST523-A-2 [Ventral]
Macrocheles penicilliger
BIN URI: BOLD:ADA2526

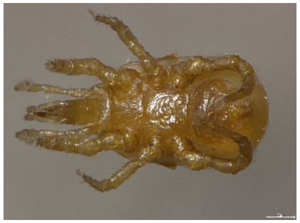

[124] UA-NST523-A-1 [Ventral]
Macrocheles penicilliger
BIN URI: BOLD:ADA2526

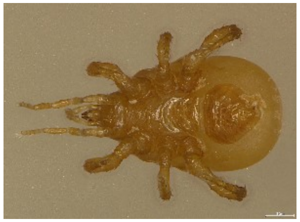

[125] UA-NST523-A-3 [Ventral]
Macrocheles penicilliger
BIN URI: BOLD:ADA2526

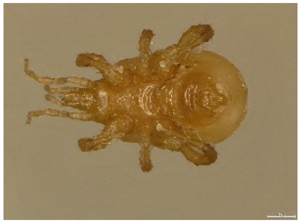

[126] UA-NST523-A-5 [Ventral]
Macrocheles penicilliger
BIN URI: BOLD:ADA2526

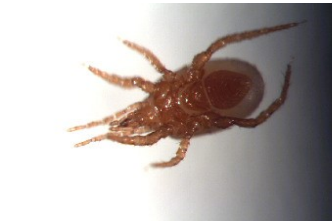

[127] POK0ME701 [Ventral]
Macrocheles matrius
BIN URI: BOLD:ADA1248

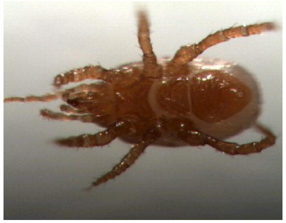

[128] POK0ME702 [Ventral]
Macrocheles matrius
BIN URI: BOLD:ADA1248

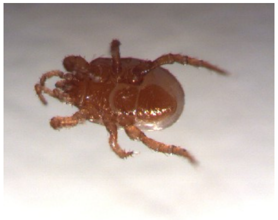

[129] POK0ME703 [Ventral]
Macrocheles matrius
BIN URI: BOLD:ADA1248

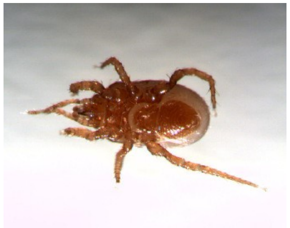

[130] POK0ME704 [Ventral]
Macrocheles matrius
BIN URI: BOLD:ADA1248

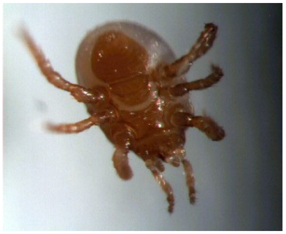

[131] POK0ME705 [Ventral]
Macrocheles matrius
BIN URI: BOLD:ADA1248

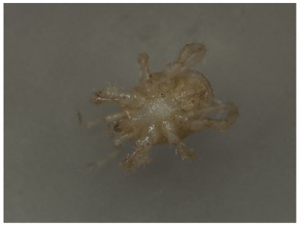

[132] HUJINVACA277 [Ventral]
Macrocheles penicilliger
BIN URI: BOLD:ADA2580

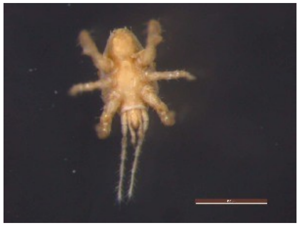

[133] MZNA442744 [Ventral]
Macrocheles muscaedomesticae
BIN URI: BOLD:ABY2066

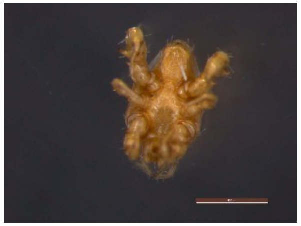

[134] MZNA442745 [Ventral]
Macrocheles muscaedomesticae
BIN URI: BOLD:ABY2066

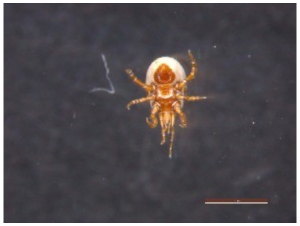

[135] MZNA442731 [Ventral]
Macrocheles muscaedomesticae
BIN URI: BOLD:ABY2066

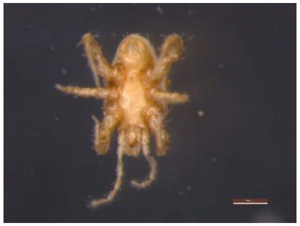

[136] MZNA442742 [Ventral]
Macrocheles muscaedomesticae
BIN URI: BOLD:ABY2066

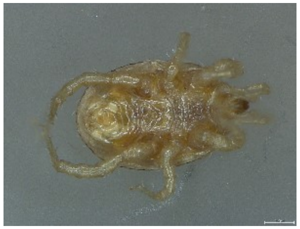

[137] UA-NST404-C-1 [Ventral]
Macrocheles muscaedomesticae
BIN URI: BOLD:ABY2066

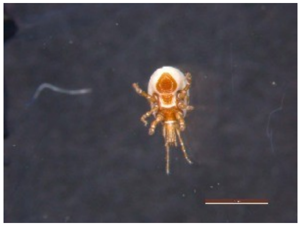

[138] MZNA442730 [Ventral]
Macrocheles muscaedomesticae
BIN URI: BOLD:ABY2066

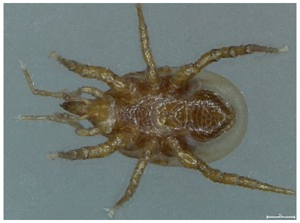

[139] UA-NST404-B-5 [Ventral]
Macrocheles muscaedomesticae
BIN URI: BOLD:ABY2066

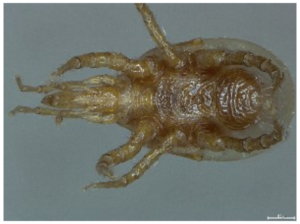

[140] UA-NST404-B-4 [Ventral]
Macrocheles muscaedomesticae
BIN URI: BOLD:ABY2066

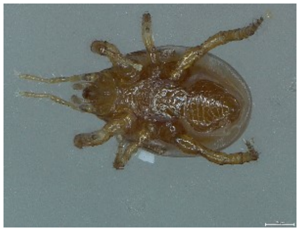

[141] UA-NST404-B-3 [Ventral]
Macrocheles muscaedomesticae
BIN URI: BOLD:ABY2066

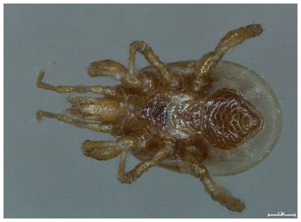

[142] UA-NST404-B-2 [Ventral]
Macrocheles muscaedomesticae
BIN URI: BOLD:ABY2066

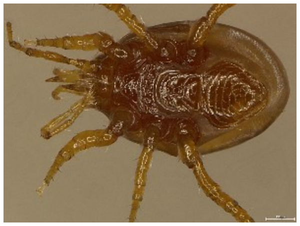

[143] UA-NST404-B-1 [Ventral]
Macrocheles muscaedomesticae
BIN URI: BOLD:ABY2066

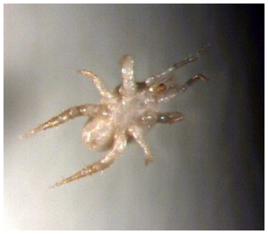

[144] LHU1ME901 [Ventral]
Macrocheles muscaedomesticae
BIN URI: BOLD:ABY2066

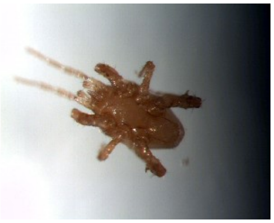

[145] LHU1ME801 [Ventral]
Macrocheles muscaedomesticae
BIN URI: BOLD:ABY2066

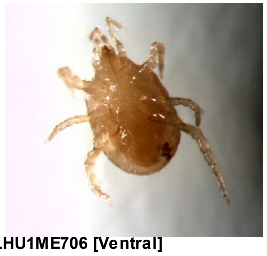

[146] LHU1ME706 [Ventral]
Macrocheles muscaedomesticae
BIN URI: BOLD:ABY2066

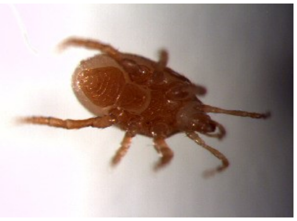

[147] LHU1ME705 [Ventral]
Macrocheles muscaedomesticae
BIN URI: BOLD:ABY2066

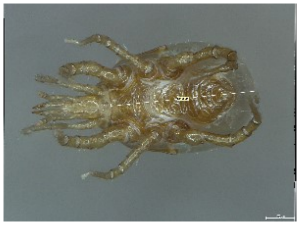

[148] UA-NST463-A [Ventral]
Macrocheles muscaedomesticae
BIN URI: BOLD:ABY2066

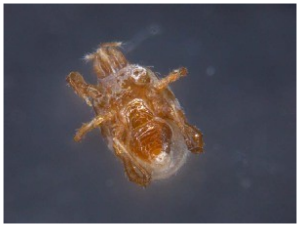

[149] MZNA442826 [Ventral]
Macrocheles muscaedomesticae
BIN URI: BOLD:ABY2066

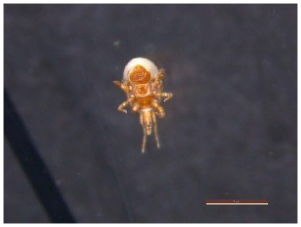

[150] MZNA442732 [Ventral]
Macrocheles muscaedomesticae
BIN URI: BOLD:ABY2066

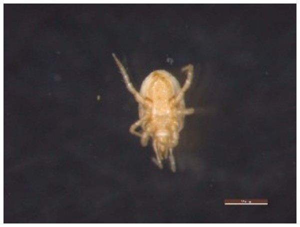

[151] MZNA442733 [Ventral]
Macrocheles muscaedomesticae
BIN URI: BOLD:ABY2066

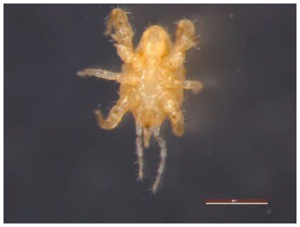

[152] MZNA442743 [Ventral]
Macrocheles muscaedomesticae
BIN URI: BOLD:ABY2066

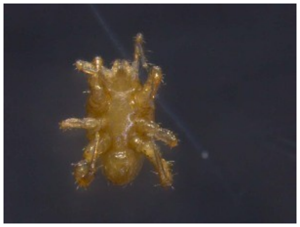

[153] MZNA442890 [Ventral]
Macrocheles muscaedomesticae
BIN URI: BOLD:ABY2066

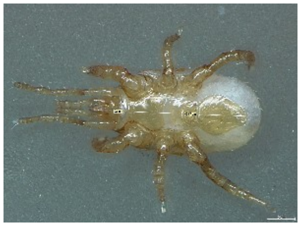

[154] HUJINVACA21 [Ventral]
Macrocheles merdarius
BIN URI: BOLD:ADA1133

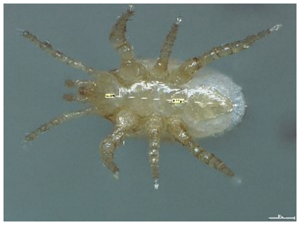

[155] HUJINVACA20 [Ventral]
Macrocheles merdarius
BIN URI: BOLD:ADA1133

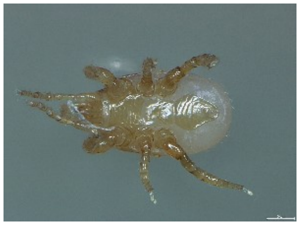

[156] HUJINVACA19 [Ventral]
Macrocheles merdarius
BIN URI: BOLD:ADA1133

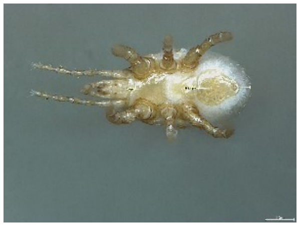

[157] HUJINVACA22 [Ventral]
Macrocheles merdarius
BIN URI: BOLD:ADA1133

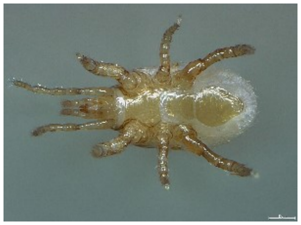

[158] HUJINVACA23 [Ventral]
Macrocheles merdarius
BIN URI: BOLD:ADA1133

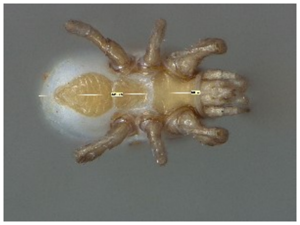

[159] HUJINVACA133 [Ventral]
Macrocheles merdarius
BIN URI: BOLD:ADA1133

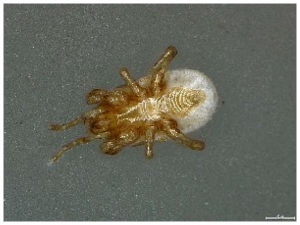

[160] HUJINVACA296 [Ventral]
Macrocheles merdarius
BIN URI: BOLD:ADA1133

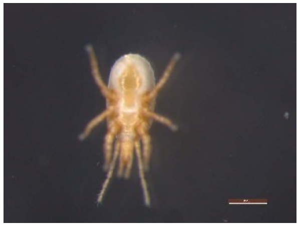

[161] MZNA442739 [Ventral]
Macrocheles merdarius

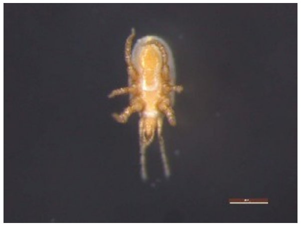

[162] MZNA442740 [Ventral]
Macrocheles merdarius
BIN URI: BOLD:ADA1540

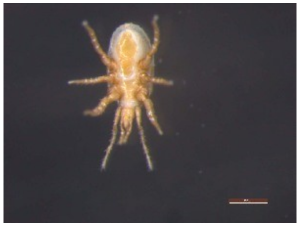

[163] MZNA442741 [Ventral]
Macrocheles merdarius
BIN URI: BOLD:ADA1540

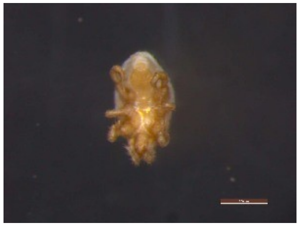

[164] MZNA442735 [Ventral]
Macrocheles merdarius
BIN URI: BOLD:ADA1540

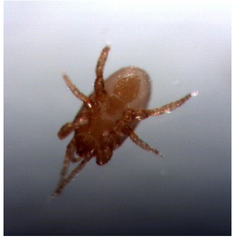

[165] POK0ME601 [Ventral]
Macrocheles merdarius
BIN URI: BOLD:ADA1540

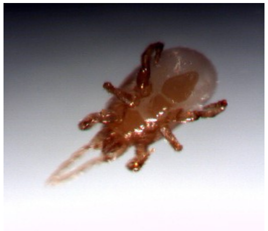

[166] POD0ME602 [Ventral]
Macrocheles merdarius
BIN URI: BOLD:ADA1540

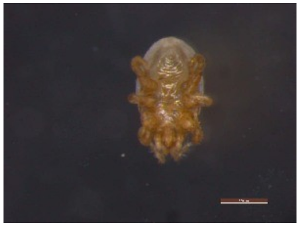

[167] MZNA442734 [Ventral]
Macrocheles merdarius
BIN URI: BOLD:ADA1540

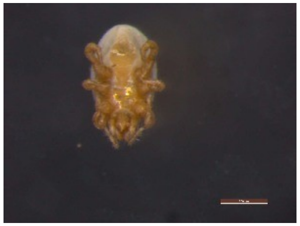

[168] MZNA442736 [Ventral]
Macrocheles merdarius
BIN URI: BOLD:ADA1540

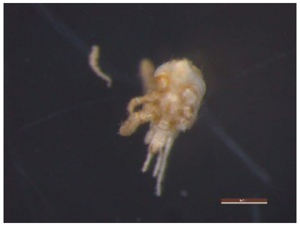

[169] MZNA442746 [Ventral]
Macrocheles merdarius
BIN URI: BOLD:ADA1540

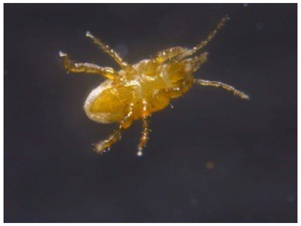

[170] MZNA442868 [Ventral]
Macrocheles scutatiformis
BIN URI: BOLD:AAH6540

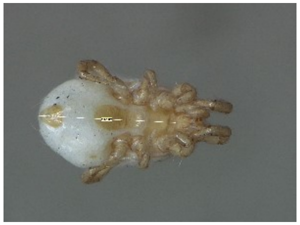

[171] HUJINVACA137 [Ventral]
Androlaelaps
BIN URI: BOLD:ADA1134

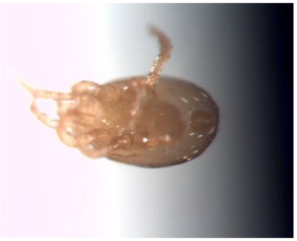

[172] CON6ME201 [Ventral]
Androlaelaps casalis
BIN URI: BOLD:ADA2853

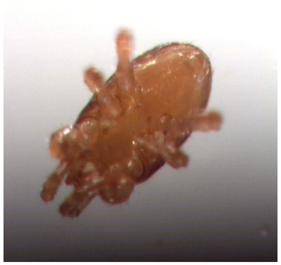

[173] BER2ME302 [Ventral]
Androlaelaps casalis
BIN URI: BOLD:ADA2853

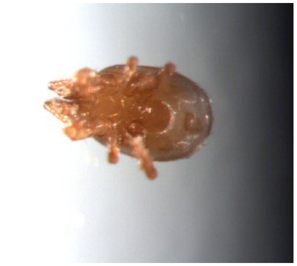

[174] BER2ME203 [Ventral]
Androlaelaps casalis
BIN URI: BOLD:ADA2853

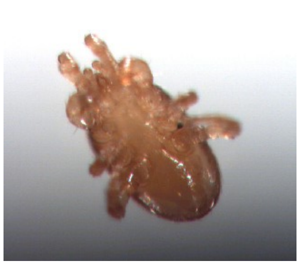

[175] BER2ME301 [Ventral]
Androlaelaps casalis
BIN URI: BOLD:ADA2853

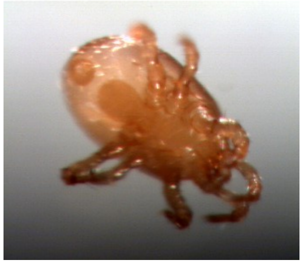

[176] BER2ME205 [Ventral]
Androlaelaps casalis
BIN URI: BOLD:ADA2853

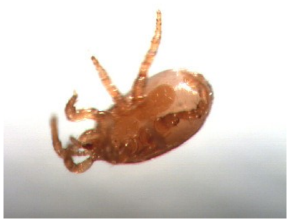

[177] BER1ME303 [Ventral]
Androlaelaps casalis
BIN URI: BOLD:ADA2853

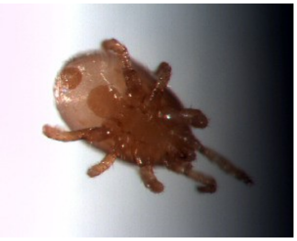

[178] BER1ME304 [Ventral]
Androlaelaps casalis
BIN URI: BOLD:ADA2853

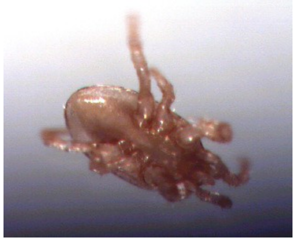

[179] CON6ME601 [Ventral]
Androlaelaps casalis
BIN URI: BOLD:ADA2853

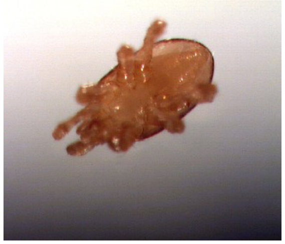

[180] CON6ME602 [Ventral]
Androlaelaps casalis
BIN URI: BOLD:ADA2853

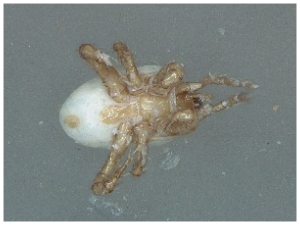

[181] HUJINVACA84 [Ventral]
Gaeolaelaps aculeifer
BIN URI: BOLD:ABW8110

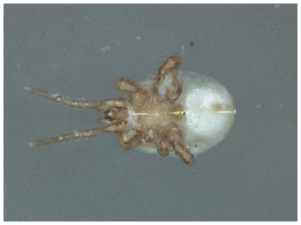

[182] HUJINVACA86 [Ventral]
Gaeolaelaps aculeifer
BIN URI: BOLD:ABW8110

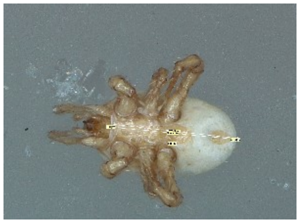

[183] HUJINVACA87 [Ventral]
Gaeolaelaps aculeifer
BIN URI: BOLD:ABW8110

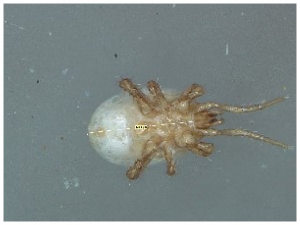

[184] HUJINVACA88 [Ventral]
Gaeolaelaps aculeifer
BIN URI: BOLD:ABW8110

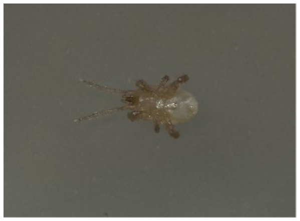

[185] HUJINVACA271 [Ventral]
Gaeolaelaps aculeifer
BIN URI: BOLD:ABW8110

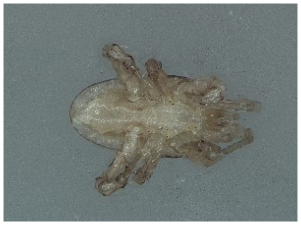

[186] HUJINVACA166 [Ventral]
Laelapidae

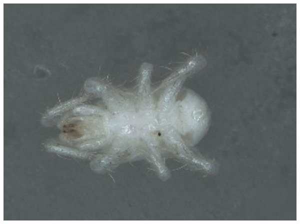

[187] HUJINVACA172 [Ventral]
Laelapidae

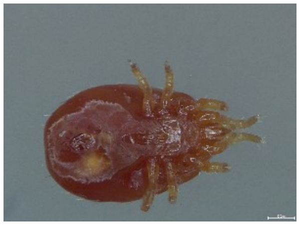

[188] HUJINVACA12 [Ventral]
Ornithonyssus sylviarum
BIN URI: BOLD:ADA5460

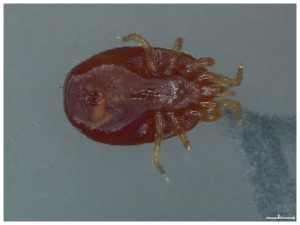

[189] HUJINVACA13 [Ventral]
Ornithonyssus sylviarum
BIN URI: BOLD:ADA5460

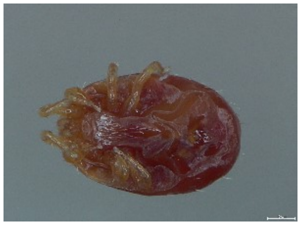

[190] HUJINVACA11 [Ventral]
Ornithonyssus sylviarum
BIN URI: BOLD:ADA5460

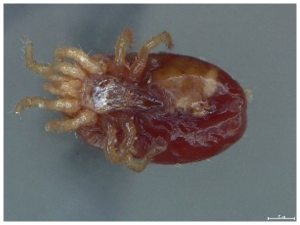

[191] HUJINVACA14 [Ventral]
Ornithonyssus sylviarum
BIN URI: BOLD:ADA5460

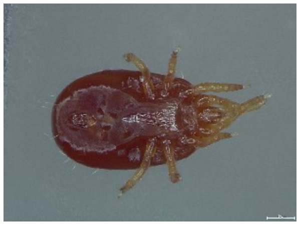

[192] HUJINVACA15 [Ventral]
Ornithonyssus sylviarum
BIN URI: BOLD:ADA5460

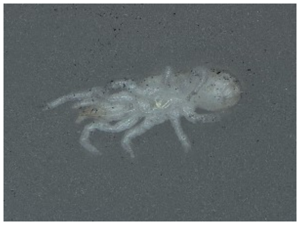

[193] HUJINVACA176 [Ventral]
Rhodacarellus silesiacus
BIN URI: BOLD:ADA2698

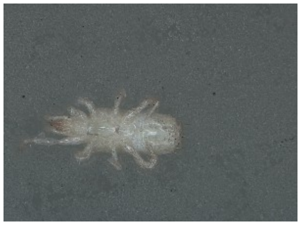

[194] HUJINVACA171 [Ventral]
Rhodacarellus silesiacus
BIN URI: BOLD:ADA1247

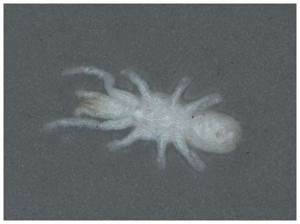

[195] HUJINVACA177 [Ventral]
Rhodacarellus silesiacus
BIN URI: BOLD:ADA1247

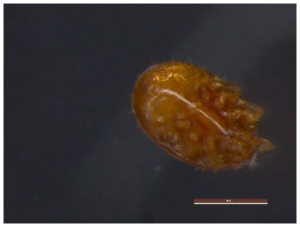

[196] MZNA442895 [Ventral]
Trematuridae
BIN URI: BOLD:ADG0003

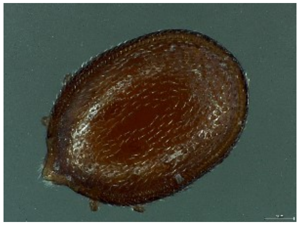

[197] HUJINVACA75 [Dorsal]
Trichouropoda ovalis
BIN URI: BOLD:ADA4889

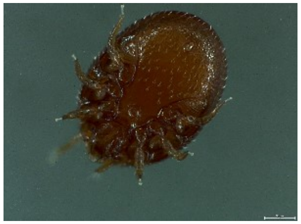

[198] HUJINVACA74 [Ventral]
Trichouropoda ovalis
BIN URI: BOLD:ADA4889

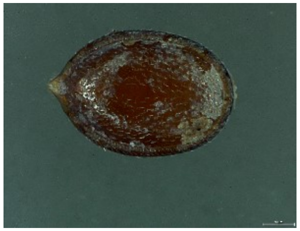

[199] HUJINVACA73 [Dorsal]
Trichouropoda ovalis
BIN URI: BOLD:ADA4889

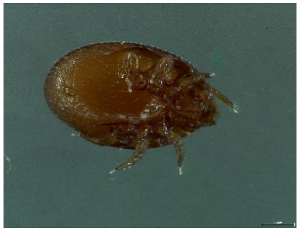

[200] HUJINVACA76 [Ventral]
Trichouropoda ovalis
BIN URI: BOLD:ADA4889

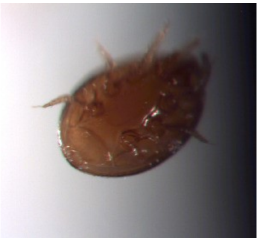

[201] PAP1UR205 [Ventral]
Uroobovella marginata
BIN URI: BOLD:ADA2195

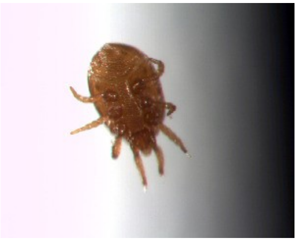

[202] PAP1UR105 [Ventral]
Uroobovella fimicola
BIN URI: BOLD:ACM5222

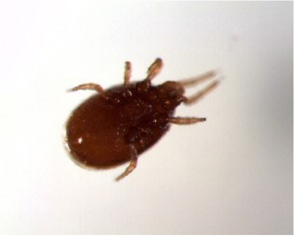

[203] GER3UR102 [Ventral]
Uroobovella fimicola
BIN URI: BOLD:ACM5222

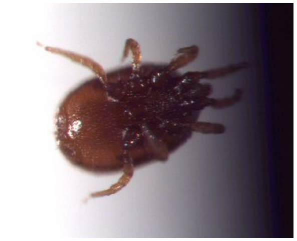

[204] GER2UR103 [Ventra]
Uroobovella fimicola
BIN URI: BOLD:ACM5222

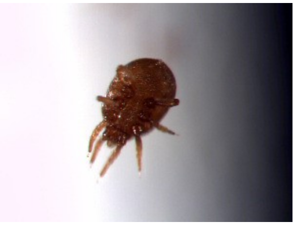

[205] GER2UR104 [Ventral]
Uroobovella fimicola
BIN URI: BOLD:ACM5222

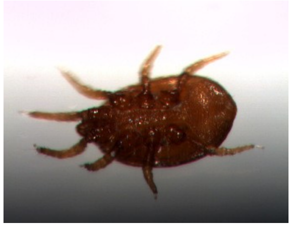

[206] GIR2UR106 [Ventral]
Uroobovella fimicola
BIN URI: BOLD:ACM5222

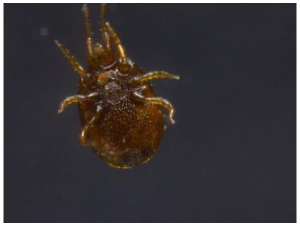

[207] MZNA442852 [Ventral]
Uroobovella fimicola
BIN URI: BOLD:ACM5222

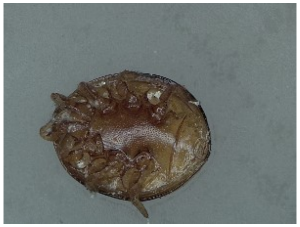

[208] HUJINVACA161 [Ventral]
Trichouropoda orbicularis
BIN URI: BOLD:ADA2194

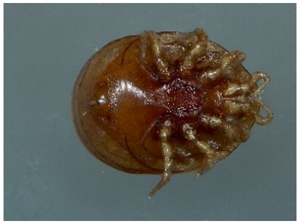

[209] HUJINVACA223 [Ventral]
Trichouropoda orbicularis
BIN URI: BOLD:ADA2194

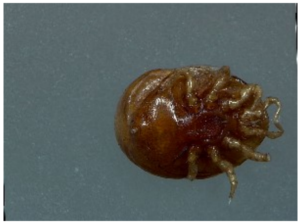

[210] HUJINVACA224 [Ventral]
Trichouropoda orbicularis
BIN URI: BOLD:ADA2194

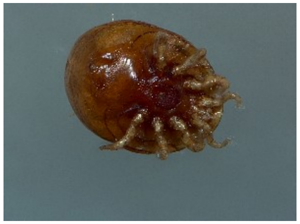

[211] HUJINVACA225 [Ventral]
Trichouropoda orbicularis
BIN URI: BOLD:ADA2194

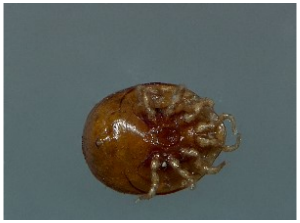

[212] HUJINVACA226 [Ventral]
Uropoda orbicularis
BIN URI: BOLD:ADA2406

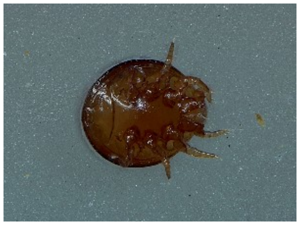

[213] HUJINVACA227 [Ventral]
Uropoda orbicularis
BIN URI: BOLD:ADA2406

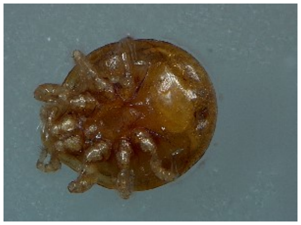

[214] HUJINVACA229 [Ventral]
Uropoda orbicularis
BIN URI: BOLD:ADA2406

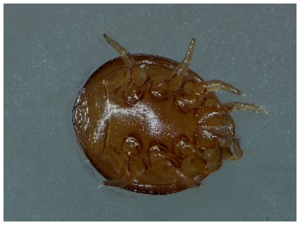

[215] HUJINVACA230 [Ventral]
Uropoda orbicularis
BIN URI: BOLD:ADA2406

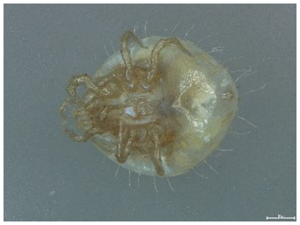

[216] UA-NST313-A-1 [Ventral]
Uroseius
BIN URI: BOLD:ADB0621

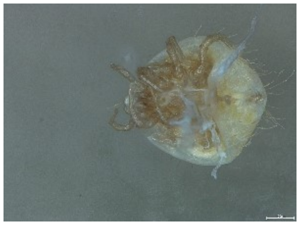

[217] UA-NST313-A-3 [Ventral]
Uroseius
BIN URI: BOLD:ADB0621

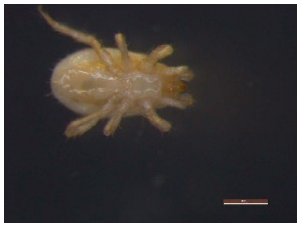

[218] MZNA442899 [Ventral]
Parasitus hyalinus
BIN URI: BOLD:ABW8099

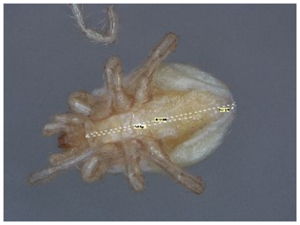

[219] HUJINVACA91 [Ventral]
Parasitus hyalinus
BIN URI: BOLD:ABW8099

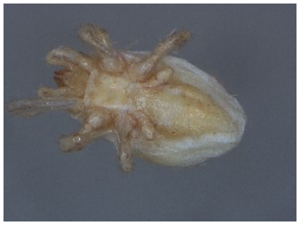

[220] HUJINVACA92 [Ventral]
Parasitus hyalinus
BIN URI: BOLD:ABW8099

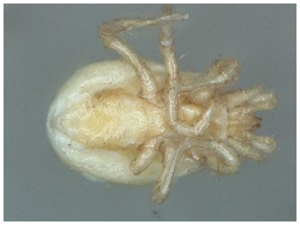

[221] HUJINVACA89 [Ventral]
Parasitus hyalinus
BIN URI: BOLD:ABW8099

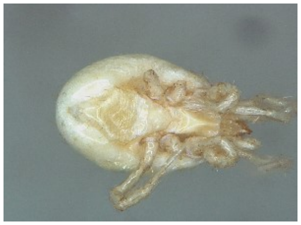

[222] HUJINVACA90 [Ventral]
Parasitus hyalinus
BIN URI: BOLD:ABW8099

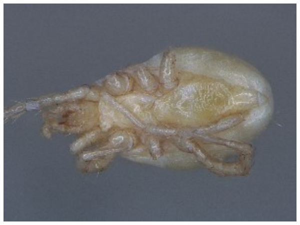

[223] HUJINVACA93 [Vental]r
Parasitus hyalinus
BIN URI: BOLD:ABW8099

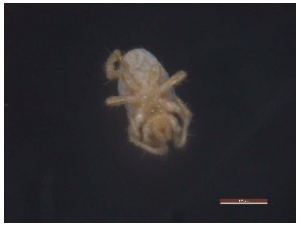

[224] MZNA442785 [Ventral]
Parasitus hyalinus
BIN URI: BOLD:ABW8099

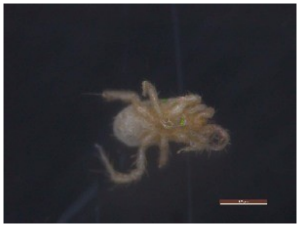

[225] MZNA442786 [Ventral]
Parasitus hyalinus
BIN URI: BOLD:ABW8099

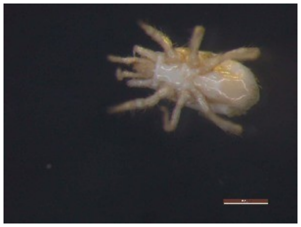

[226] MZNA442896 [Ventral]
Parasitus hyalinus
BIN URI: BOLD:ABW8099

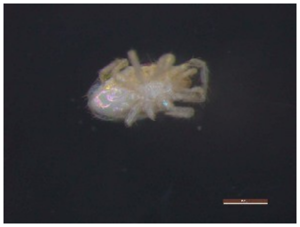

[227] MZNA442897 [Ventral]
Parasitus hyalinus
BIN URI: BOLD:ABW8099

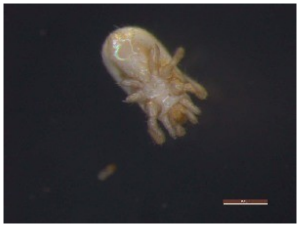

[228] MZNA442898 [Ventral]
Parasitus hyalinus
BIN URI: BOLD:ABW8099

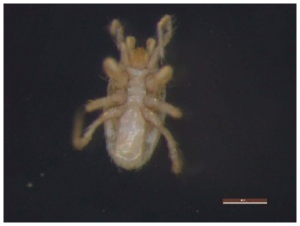

[229] MZNA442900 [Ventral]
Parasitus hyalinus
BIN URI: BOLD:ABW8099

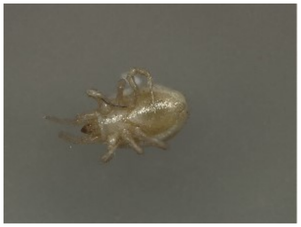

[230] HUJINVACA269 [Ventral]
Coleogamasus
BIN URI: BOLD:ADA2804

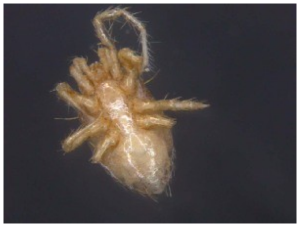

[231] MZNA442838 [Ventral]
Parasitus fimetorum
BIN URI: BOLD:ADG0220

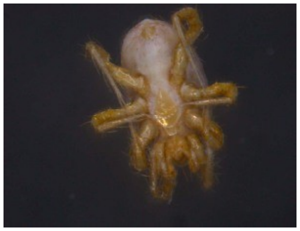

[232] MZNA442782 [Ventral]
Parasitus fimetorum
BIN URI: BOLD:ADG0220

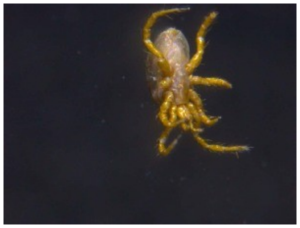

[233] MZNA442875 [Ventral]
Parasitus fimetorum
BIN URI: BOLD:ADG0220

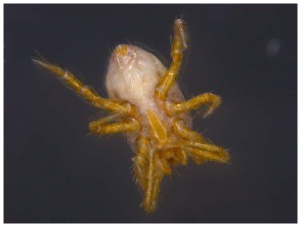

[234] MZNA442839 [Ventral]
Parasitus fimetorum
BIN URI: BOLD:ADG0220

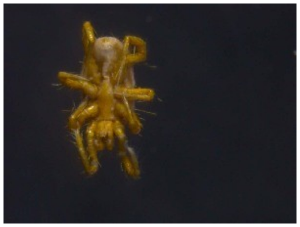

[235] MZNA442879 [Ventral]
Parasitus fimetorum
BIN URI: BOLD:ADG0220

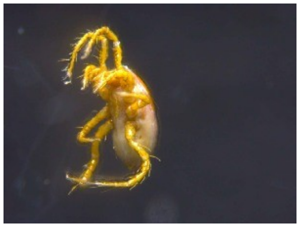

[236] MZNA442872 [Ventral]
Parasitus fimetorum
BIN URI: BOLD:ADG0220

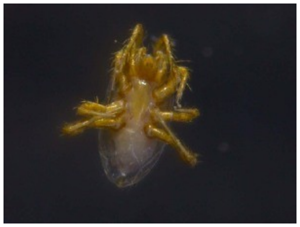

[237] MZNA442874 [Ventral]
Parasitus fimetorum

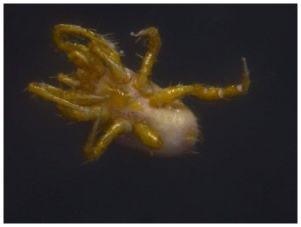

[238] MZNA442881 [Ventral]
Parasitus fimetorum
BIN URI: BOLD:ADG0220

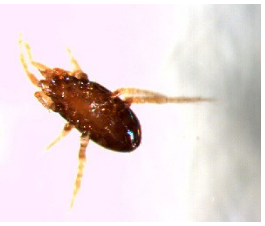

[239] GIR2ME501 [Ventral]
Parasitus fimetorum
BIN URI: BOLD:AAF9222

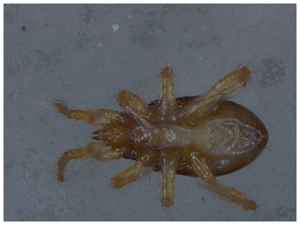

[240] HUJINVACA104 [Ventral]
Parasitus fimetorum
BIN URI: BOLD:AAF9222

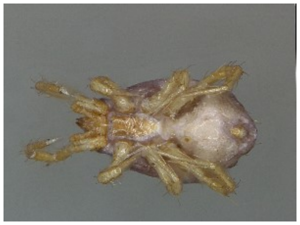

[241] HUJINVACA119 [Ventral]
Parasitus fimetorum
BIN URI: BOLD:AAF9222

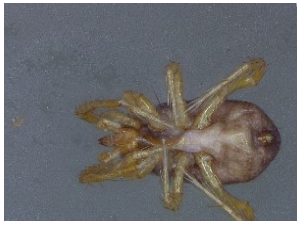

[242] HUJINVACA108 [Ventral]
Parasitus fimetorum
BIN URI: BOLD:AAF9222

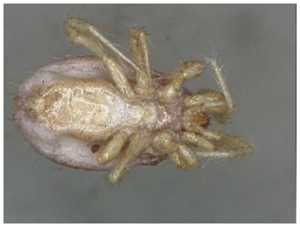

[243] HUJINVACA114 [Ventral]
Parasitus fimetorum
BIN URI: BOLD:AAF9222

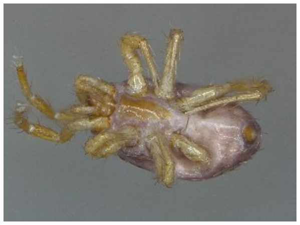

[244] HUJINVACA122 [Ventral]
Parasitus fimetorum
BIN URI: BOLD:AAF9222

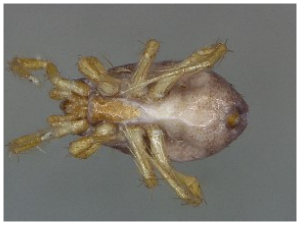

[245] HUJINVACA121 [Ventral]
Parasitus fimetorum
BIN URI: BOLD:AAF9222

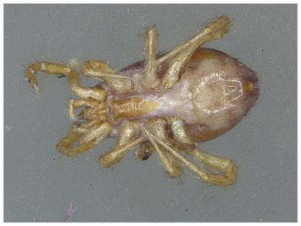

[246] HUJINVACA123 [Ventral]
Parasitus fimetorum
BIN URI: BOLD:AAF9222

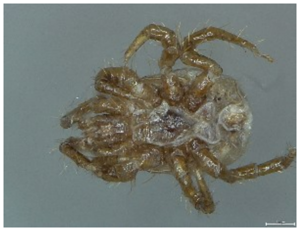

[247] UA-NST460-C-4 [Ventral]
Poecilochirus carabi
BIN URI: BOLD:ADA9775

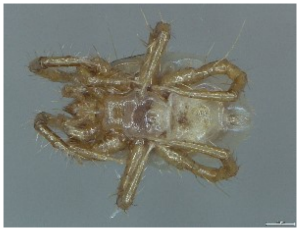

[248] UA-NST512-B-2 [Ventral]
Poecilochirus carabi
BIN URI: BOLD:ADA9775

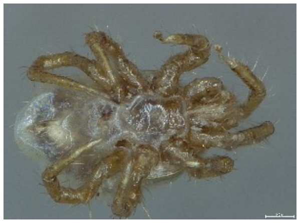

[249] UA-NST460-C-1 [Ventral]
Poecilochirus carabi
BIN URI: BOLD:ADA9775

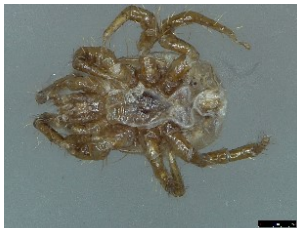

[250] UA-NST460-C-2 [Ventral]
Poecilochirus carabi
BIN URI: BOLD:ADA9775

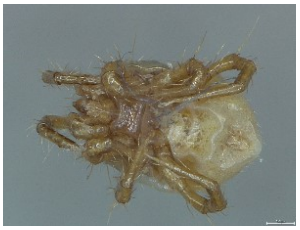

[251] UA-NST512-B-1 [Ventral]
Poecilochirus carabi
BIN URI: BOLD:ADA9775

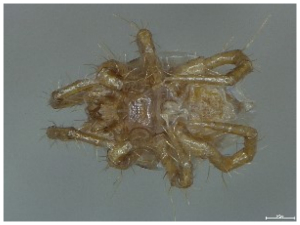

[252] UA-NST512-B-3 [Ventral]
Poecilochirus carabi
BIN URI: BOLD:ADA9775

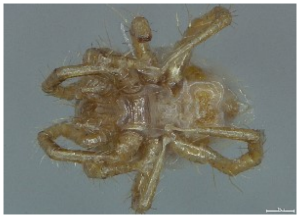
[253] UA-NST512-B-4 [Ventral]
Poecilochirus carabi
BIN URI: BOLD:ADA9775

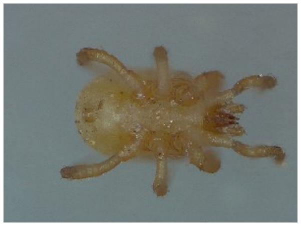
[254] HUJINVACA205 [Ventral]
Gamasodes spiniger
BIN URI: BOLD:ADA2803

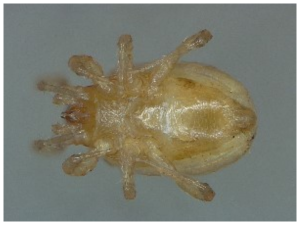
[255] HUJINVACA199 [Ventral]
Gamasodes spiniger
BIN URI: BOLD:ADA2803

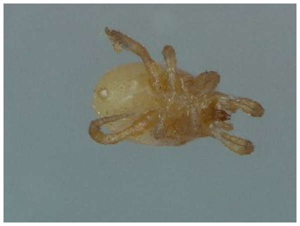
[256] HUJINVACA206 [Ventral]
Gamasodes spiniger
BIN URI: BOLD:ADA2803

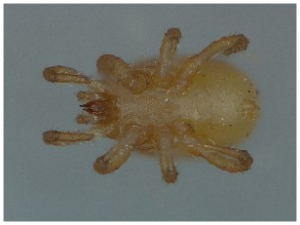
[257] HUJINVACA202 [Ventral]
Gamasodes spiniger
BIN URI: BOLD:ADA2803

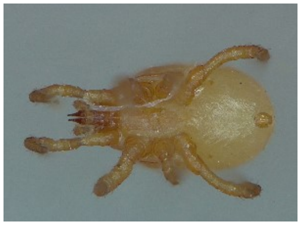
[258] HUJINVACA203 [Ventral]
Gamasodes spiniger
BIN URI: BOLD:ADA2803

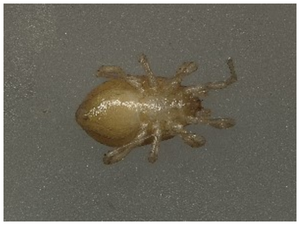
[259] HUJINVACA274 [Ventral]
Gamasodes spiniger
BIN URI: BOLD:ADA2803

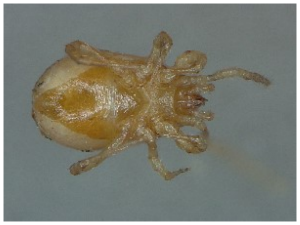
[260] HUJINVACA198 [Ventral]
Gamasodes spiniger
BIN URI: BOLD:ADA2803

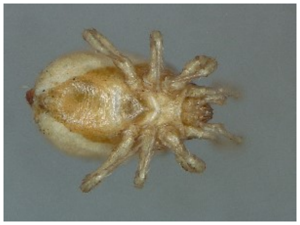
[261] HUJINVACA200 [Ventral]
Gamasodes spiniger
BIN URI: BOLD:ADA2803

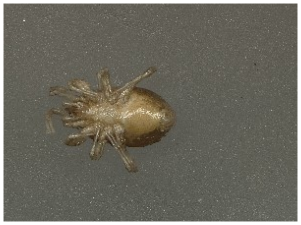
[262] HUJINVACA275 [Ventral]
Gamasodes spiniger
BIN URI: BOLD:ADA2803

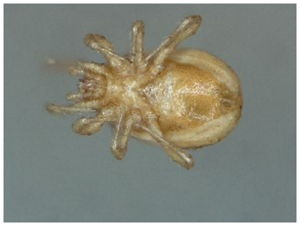
[263] HUJINVACA201 [Ventral]
Gamasodes spiniger
BIN URI: BOLD:ADA2803

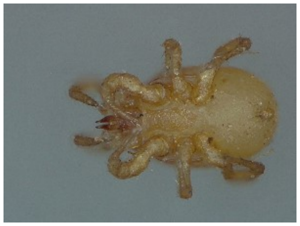
[264] HUJINVACA204 [Ventral]
Gamasodes spiniger
BIN URI: BOLD:ADA2803

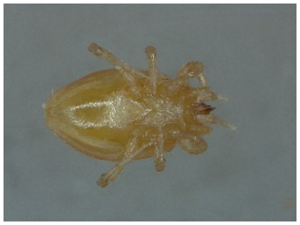
[265] HUJINVACA197 [Ventral]
Gamasodes spiniger
BIN URI: BOLD:ADA2803

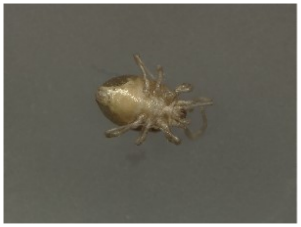
[266] HUJINVACA276 [Ventral]
Gamasodes spiniger
BIN URI: BOLD:ADA2803

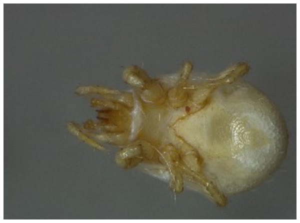
[267] HUJINVACA127 [Ventral]
Vulgarogamasus burchanensis
BIN URI: BOLD:ADA1245

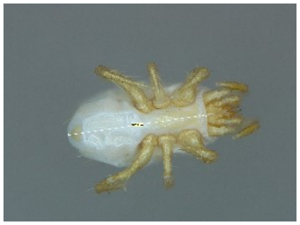
[268] HUJINVACA138 [Ventral]
Vulgarogamasus burchanensis
BIN URI: BOLD:ADA1245

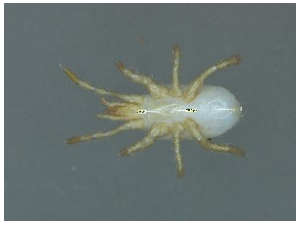
[269] HUJINVACA139 [Ventral]
Vulgarogamasus burchanensis
BIN URI: BOLD:ADA1245

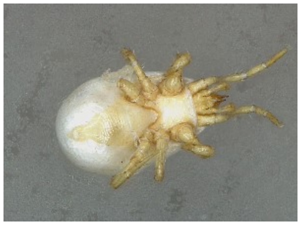
[270] HUJINVACA125 [Ventral]
Vulgarogamasus burchanensis
BIN URI: BOLD:ADA1245

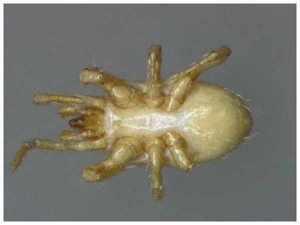
[271] HUJINVACA132 [Ventral]
Vulgarogamasus burchanensis
BIN URI: BOLD:ADA1245

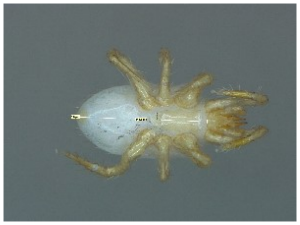
[272] HUJINVACA140 [Ventral]
Vulgarogamasus burchanensis
BIN URI: BOLD:ADA1245

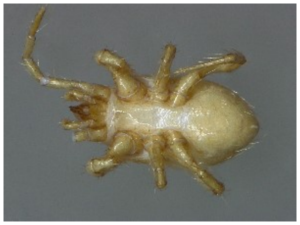

[273] HUJINVACA129 [Ventral]
Vulgarogamasus burchanensis
BIN URI: BOLD:ADA1245

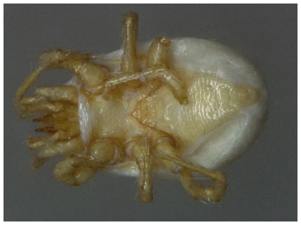

[274] HUJINVACA128 [Ventral]
Vulgarogamasus burchanensis
BIN URI: BOLD:ADA1245

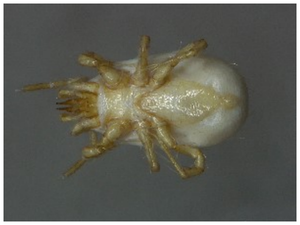

[275] HUJINVACA126 [Ventral]
Vulgarogamasus burchanensis
BIN URI: BOLD:ADA1245

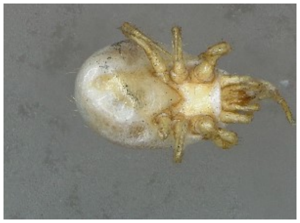

[276] HUJINVACA124 [Ventral]
Vulgarogamasus burchanensis
BIN URI: BOLD:ADA1245

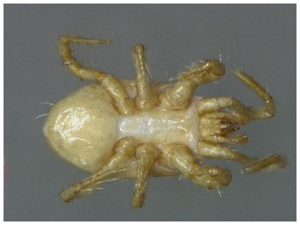

[277] HUJINVACA130 [Ventral]
Vulgarogamasus burchanensis
BIN URI: BOLD:ADA1245

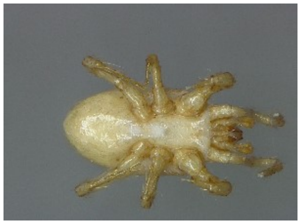

[278] HUJINVACA131 [Ventral]
Vulgarogamasus burchanensis
BIN URI: BOLD:ADA1245

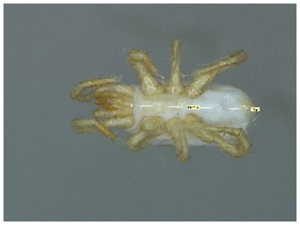

[279] HUJINVACA141 [Ventral]
Vulgarogamasus burchanensis
BIN URI: BOLD:ADA1245

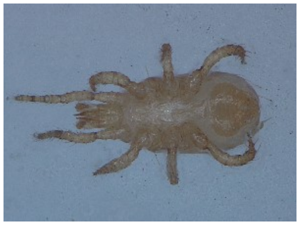

[280] HUJINVACA101 [Ventral]
Lasioseius fimetorum
BIN URI: BOLD:ADA1359

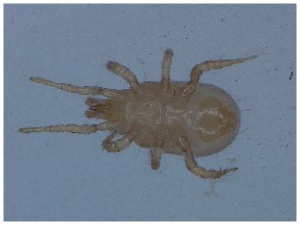

[281] HUJINVACA102 [Ventral]
Lasioseius fimetorum
BIN URI: BOLD:ADA1359

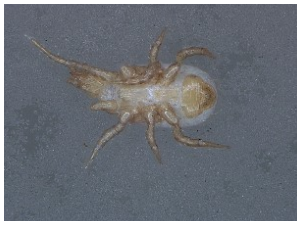

[282] HUJINVACA99 [Ventral]
Lasioseius fimetorum
BIN URI: BOLD:ADA1359

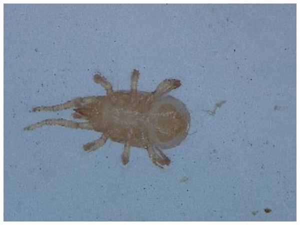

[283] HUJINVACA100 [Ventral]
Lasioseius fimetorum
BIN URI: BOLD:ADA1359

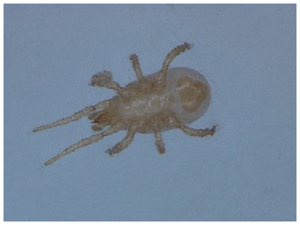

[284] HUJINVACA103 [Ventral]
Lasioseius fimetorum
BIN URI: BOLD:ADA1359

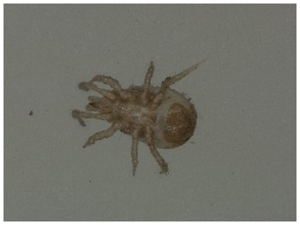

[285] HUJINVACA262 [Ventral]
Lasioseius fimetorum
BIN URI: BOLD:ADA2196

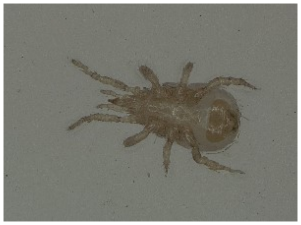

[286] HUJINVACA261 [Ventral]
Lasioseius fimetorum
BIN URI: BOLD:ADA2196

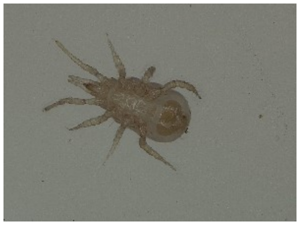

[287] HUJINVACA260 [Ventral]
Lasioseius fimetorum
BIN URI: BOLD:ADA2196

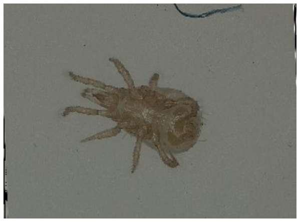

[288] HUJINVACA263 [Ventral]
Lasioseius fimetorum

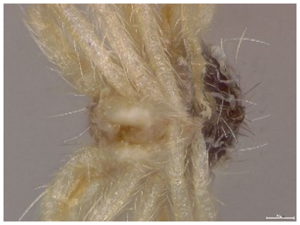

[289] UA-NST460-W-3 [Ventral]
Anystidae
BIN URI: BOLD:AAF9236

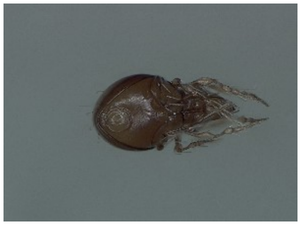

[290] HUJINVACA158 [Ventral]
Oppiidae
BIN URI: BOLD:ADA2122

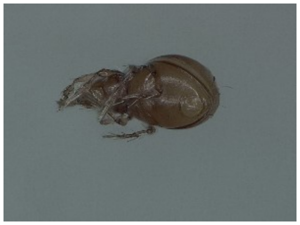

[291] HUJINVACA157 [Ventral]
Oppiidae
BIN URI: BOLD:ADA2122

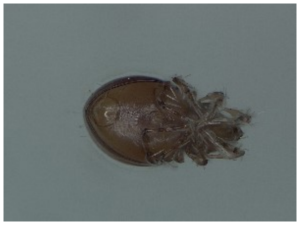

[292] HUJINVACA156 [Ventral]
Oppiidae
BIN URI: BOLD:ADA2122

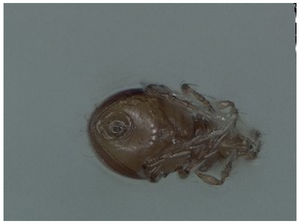

[293] HUJINVACA159 [Ventral]
Oppiidae
BIN URI: BOLD:ADA2122

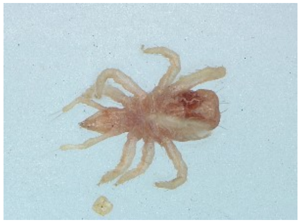

[294] HUJINVACA212 [Ventral]
Bdellidae
BIN URI: BOLD:ADA1736

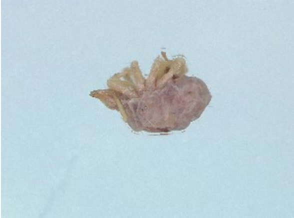

[295] HUJINVACA213 [Ventral]
Bdellidae
BIN URI: BOLD:ADA1567

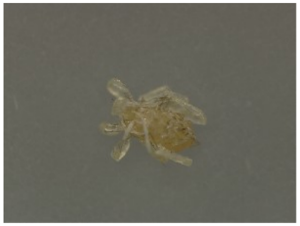

[296] HUJINVACA266 [Ventral]
Erythracaridae
BIN URI: BOLD:ADA2655

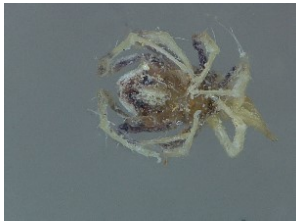

[297] HUJINVACA185 [Ventral]
Bdellidae
BIN URI: BOLD:ADA2274

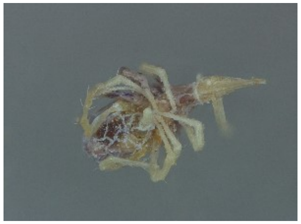

[298] HUJINVACA186 [Ventral]
Bdellidae
BIN URI: BOLD:ADA2274

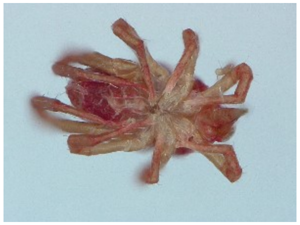

[299] HUJINVACA214 [Ventral]
Bdellidae
BIN URI: BOLD:ADA2727

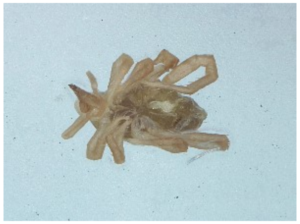

[300] HUJINVACA217 [Ventral]
Bdellidae
BIN URI: BOLD:ADA2727

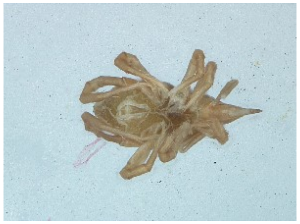

[301] HUJINVACA218 [Ventral]
Bdellidae
BIN URI: BOLD:ADA2727

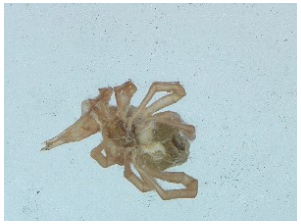

[302] HUJINVACA219 [Ventral]
Bdellidae
BIN URI: BOLD:ADA2727

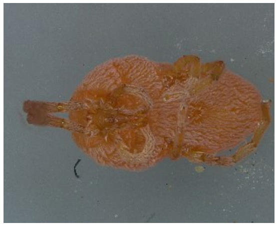

[303] HUJINVACA191 [Ventral]
Trombidiidae
BIN URI: BOLD:ADA2906

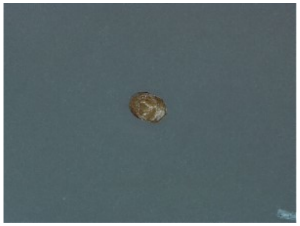

[304] HUJINVACA244 [Ventral]
Scutacarus
BIN URI: BOLD:ADA2726

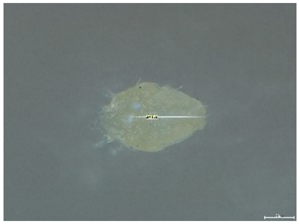

[305] HUJINVACA33 [Dorsal]
Tydeidae
BIN URI: BOLD:ADA3438

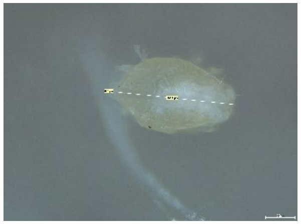

[306] HUJINVACA35 [Dorsal]
Tydeidae
BIN URI: BOLD:ADA3438

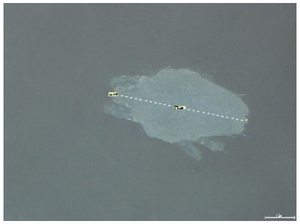

[307] HUJINVACA36 [Dorsal]
Tydeidae
BIN URI: BOLD:ADA3438
